# Supplementary material for: The phage Φ13-encoded transcriptional regulator Ltr controls phage assembly in Staphylococcus aureus
Source: Virol J. 2026 Apr 24;23:111. doi: 10.1186/s12985-026-03167-5 (PMC13123087; doi:10.1186/s12985-026-03167-5)
Supplement: Supplementary file 1 — Supplementary material 1 [file 12985_2026_3167_MOESM1_ESM.docx]

**Supplementary Information**

**
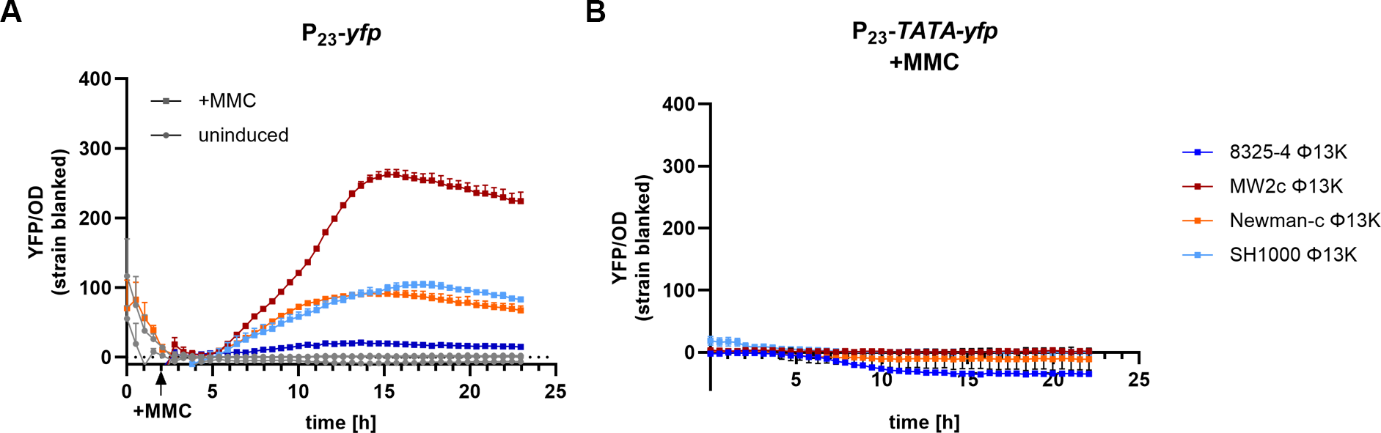
**

**Figure S1: P_23_ promoter activity measurement over time.** Strains carrying (A) the wild type (P*_23_*-*yfp*: pCG896) or (B) the TATA mutant reporter plasmid (P*_23_*-*TATA*-*yfp*: pCG910) were grown to exponential phase, induced with subinhibitory mitomycin C (MMC), and incubated for 24 h. Optical density and fluorescence were measured. Arbitrary units of fluorescence (YFP) are shown normalized to OD_600_; strain-specific background fluorescence was subtracted. (A) Comparison of strain-specific activity of the wild type P_23_ promoter under uninduced and induced (+MMC) conditions. (B) Promoter activity of P_23_ with mutated TATA-box (P_23_-*TATA*-*yfp*).


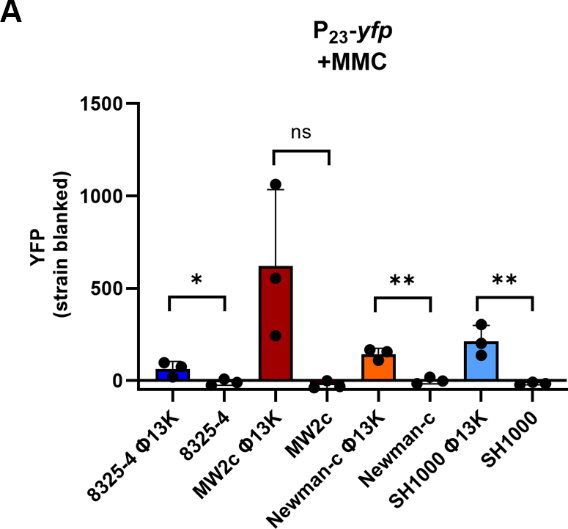


**Figure S2: P23 activity in single-lysogenic and phage-free background.** (A) Single time point measurement of P_23_ promoter activity in single-lysogens carrying Φ13K (wild type) and in phage-free strains. Strains were grown to exponential phase, induced with a subinhibitory concentration of mitomycin C (MMC) (300 ng ml^-1^), and grown for further 4 hours. Bacteria were harvested to OD_600_ of 2 and resuspended in PBS. Fluorescence was measured. Arbitrary units of fluorescence (YFP) are shown, strain-specific background fluorescence was subtracted. Data shown are mean ± SD (n = 3). Statistical significance was determined by unpaired t-tests within strains. (**p-value < 0.01, *p-value < 0.05, ns > 0.05).


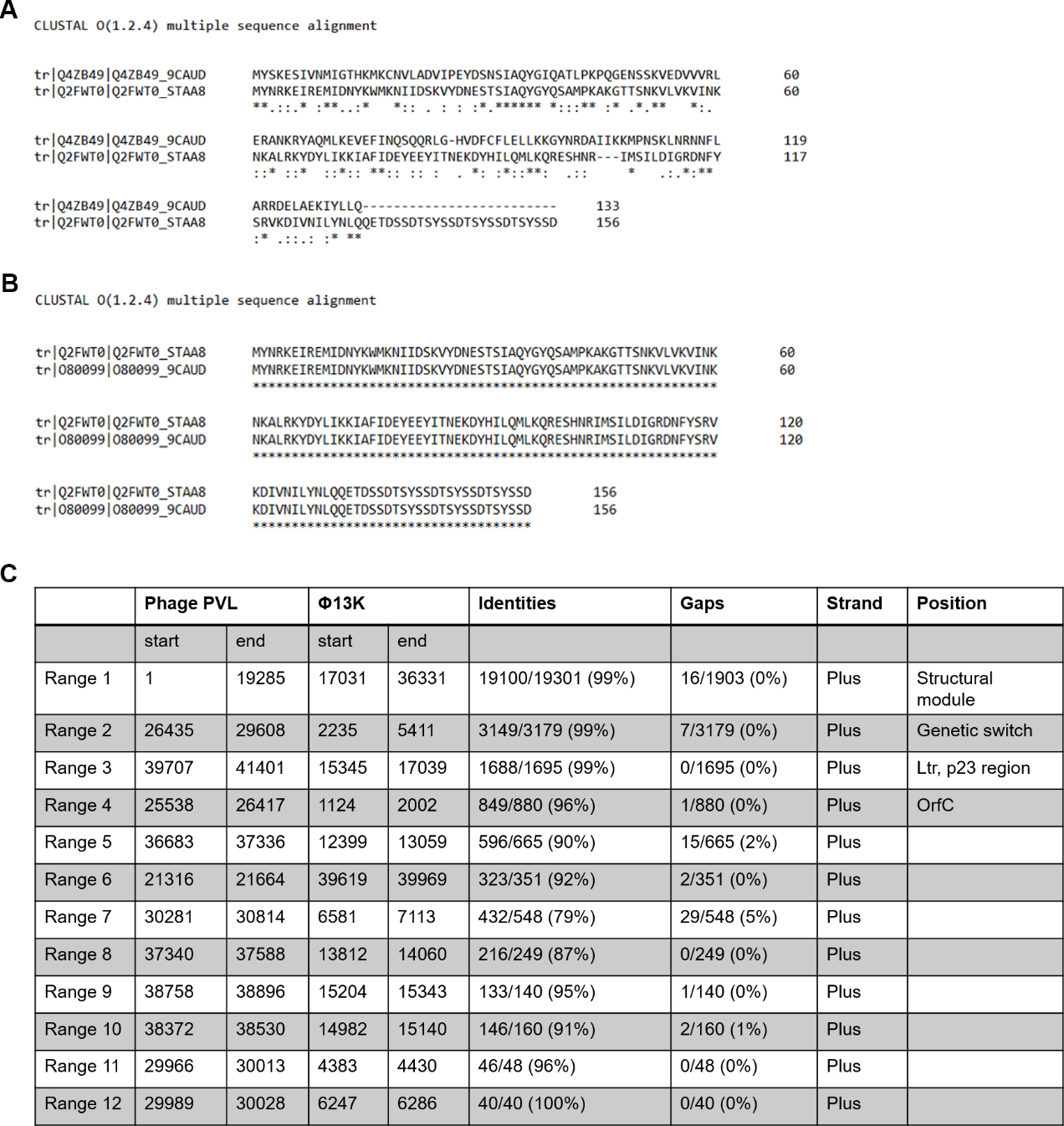


**Figure S3: Alignment of Ltrs and phage genomes** (A,B) Protein sequence alignment of putative Φ13-Ltr (*SAOUHSC_02200*, UniProt ID: Q2FWT0) and ORF34 of phage Φ55 (UniProt ID: Q4ZB49) (A) and hypothetical protein PVL_60 (UniProt ID: O80099) (B). Symbols below sequences indicate the degree of conservation, with ‘*‘ indicating fully conserved residues, ‘:‘ indicating conservation between groups of strongly similar properties (Gonnet PAM 250 > 0.5), ‘.‘ indicating conservation between groups of weakly similar properties (Gonnet PAM 250 ≤ 0.5), and a space indicating non-conserved residues. (C) Overview of nucleotide alignment results of phage Φ13K genome (see Supplement) and phage PVL genome (Accession number: NC_002321).

**Φ13K genome – nucleotide sequence**

>prophage_phi13Kan_TSS_annotated

**CCAGTTTGGATACATAGAAACCTTGTAACAACAGTATTTATTGGGTTTGGAGTCCCTAATGGGTCCCTAAATTACATACTTTCTAAAATTTTAGTTGTTTTTTTGTCCTCTTCATTAAATTTTTCTTCTAACAAATGAGAATACACGGATGTAGTTATTGCTATATTTTTATGACCTAATCTTTTAGAAATGTAATGTATAGATACACCTTTTGCTAGTAAATAAGAACAATGAGTGTGTCTTAATGCGTGCGATGTAATAATTGGTATATTATTGACTCTACAGGCTGATTTCAAAGCATTATTGATAGCCTGAAGGTTAATTATAGATCCGGCTTCTTTGAAAATGTAACCATCATAGCTAATTGCAAATGTACTTATGACGTCCATAATGTGTTTCATATCAGATTTAGCGATACTGATATATCTAGGGGAAGTATCGGTTTTTCGCTCGTCAATAAATATAGTGTTTTTCACTTGGTTGATATGCTCAATCTTTATATTTCTTGCACCACTGACACGACAACCCGTACAAATCATTATGAATAGCGCTAATGATGAACGAGTTCTCTTCTTTCTGACGTGATCTTTTAGTATTTCATATTCAGTTACCGAGATGAATTTTTCTTGTTCTGACTTCGTAGGTTTTCCGGCTTTATAATTAACTTTATAAGCGGGGTTTTTAAAAATAAGTCCATCATATAATGCGTCATCTAAAGCTGACCGAATAGCACCGTTTGTTTTTCTTATAGTTTCTTTTGCGTGTTCTTTTGAATAGTCGTTTATGAATTTCTGATAAACTTGTCTATTTATCTTTGATAACTCCATTTTACCTATTTTATGTTTTTGTATATGTTGTAATGCATTTCTATAATGACGGTAGGTATTTTCTTTAACAACAGGTTGTTTATATGTTTTAATCCAATTTTCGAAGTATTCTGCAAGAGTTATATAGTTATCTATATTAAAACCACTTCTTAACTCATTTAACTTGTCTAGTCCAGCAGAATTAGCTTCACGCTTTGTTCTAAAACCTTTCTTACGGTATCTTTTTCCTTCATGCTTAAATTCATATTGCCATTTTTTACCATCGTAACAACGTGTTTTCATGCGTTCCCTCCTCAAAATTGGCAAAAAATAATAAGGGTAGGCGGGCTACCCAAAATTTAGTACTAGGTACTAAATATGTTATAATAAAATAAAAAGTAGGTGATAAGATGACTCAATTTCTAGGGGCGCTTCTTCTTACAGGAGTTTTAGGTTACATACCATATAAATATCTAACAATGATAGGTTTAGTTAGTGAAAAAAACAAGATTATCAATACTCCTGTATTATTGATTTTTTCTATTGAAACATGTTTGATATGGTTTTATACTTTTATAATTTTTAATAATGTTGATTTAAAAAATTTGAGTTTACTTCAGTTGCTTACAGGTCTAAAAGCAAATATTTGGTTTCTAATTATTTTTGTTTTAACAGTGCTTGTATTTAATCCTTTAATTGTTAAATTCATTATCTGGTTAATTAATGAAACAAGAAAGTTTATGAATTTGGATTGTATAAGCTTATTAGACAAAAGAGACAAGTTGTTTAATAACAACGGTAAACCAGTATTTATAGTTATTAAAGACTTTGAAAACAGAATCATTGAAGAGGGTGAACTTAAAACCTATAATTCAGCTGGTAGCGATTTCGATTTACTAGAGGTTGAGCGACAAGATTTCAAAGTATCTGATTTACCGTCAAACGATGAATTGTATATTAAACATACACTTGTAGACCTTAAACAACAAATTAAATTGGATTTATATTTAATGAATGAATATTAATCTTTTTTCTTAGCTTTTTCTGATAAAGTGCTTTTTAAGTTTTCGCTGGCACCCGGCTTTTCAAAACTTTTGTTTATTGGGTTACTACGAGTAGCTTCTTGTTTTTTGTTTTTATCCGCCATAAAATTCTCACCACCATTCAACGTCTACACTTGTAGGCGTTTTTTGTTTAGTAAAATCATAATGAATCTTCTTTGGTTAACTTATCGCCATCTAATTTTTGTGAAATAAATTCCAAGTATTTACGCGCATTATGTGACGATAAATCTTTAGGTAACTCATAAGTGAATGGTTGATTACCACTAGTTAAAACTTCATATACTATAGTTTCTTTTTTATTTTGCAATTAGTTATTTTCATTATAAACTTCCTTTCAAACACTGCTGAAATAGACGTCTTTTTCAAATAAGCATGATTAATACTTCAATTCTTTAATCCACATATATTTAAAAGTGAGGTAGTAGGTAATAAATATAAGACTTAAAGTTAAGATTGCTTTTTTCATGTCAATTTCTCCTTTGTTTATATTTATATTAAAGCGCTAAATATACGTTATTAATCACAATACAACTTTGCCCATTACTTTAATATCACTAAACGAAGCGACTTTGATATCATCATACTTCGGATTTAGAGATACCAAATTAATATAGTCTTCGCATATATCTACACGCTTGATAAGACTTACTCCATCTAATACAACGAGTGCAATTGTACCATCTTTAATAGAATCTTCTTTCTTAATAAAAGCGTATGTTCCTTGTTTTAACATAGGTTCCATTGAATCACCATTAACTAAAATACAAAAATCAGCATTTGATGGCGTTTCGTCTTCTTTAAAAAATACTTCTTCATGCAATATGTCATCATATAATTCTTCTCCTATGCCAGCACCAGTTGCACCACATGCAATATACGATACTAGTTTAGACTCTTTATATCCATCTATAGAAGTGACTTTATTCTGTTCTTCCAATTGTTCATTTGCATAGTTAAGTACGTTTTCTTGGCGGGGAGGTGTGAGTTGAGAAAATATGTTATTGATTTTTGACATTATCGTTTCATCTTGACGTTCTTCATCAGGAACTCGATAAGAATCTACATCATACCCCATAAGCCACGCTTCACCAACGTTCAGAGTTTTAGAAAGTAGGTAAATTCTATCTTGGTCGGGTGATTGTACGTCGTTAATATATTGAGATAAAGTGCTTTTACTTAAAGATATACCTAGTTTCTTTTGATAAGGTTTCGATTTATTAATGATATCTACTTGTTTTAAGTTTCTTATTTTCATAATGTGTTTAAGTCTATTTGAAACTTTTTCTCTCATTTAGTGCACCTCCGTTTGATAACTTCATAATAAAGCTTGTTGAACAAAAATTCAACAAAAAAGTTCATAAATCATGAATTTTTGTATTGACTTGATTCAAAACAAGGTGTAAAGTATAGTTAAGTTCATGATACGTGAACTTGAGAGGAGGTGCTTTTATGTGTTACGACTACTCACGTTTGAGCGGGAAAATAGTAGAAAAGTATGGCACTCAGTACAATTTTGCAATTGCTATGAAATTGTCAGAGAGAAGTTTATCCTTAAAACTCAACGGTAAAGTTGGTTGGAAAGACAGTGAAATATGGAAAGCTATACAACTACTAGATATACCGGTAGAGAAAATACACTTATATTTTTTTAAAGAAAAAGTTCATGTTATATGAACTTAAGGAGGGGCACAATGGAACAAATCACGTTAACCAAAGAAGAGTGTGTCGAACAATGCATCAATAAAGACTTAAAACTTTTAGATTATCGAGTTCAACAAATTTTAGAAGGTGTTCTATCAGAAAGTACCACATACGGTGATGCAAGAAATAAATTAGAAACATTGAAAATTATTGCTGAATCTCATTTTAAAACCGAACATGCTTCAGTTATTTACAAATTAGCATTGAAAAAGTTAGACGAAAAAATCAACGCCACTCCAATTAAAGAGTGACGGAAAGGGAGGATTTTAAATGTTTAAGGTTTTAAATGATATAAAAACTTCTTTAAAAAACCATCCTTGGGGTTGGAAAGAGCACTTACCTTATTTGCTGATGTTAACTCTGTCACTTGTGGCTCTGATTCTCGGTGTTCTGTCCGCGATTCTATGATAACAGGCTTTATATAGATTCCTTTGTTGGTAGTGACTTTGATAGTCACATCCCATTCCCATATCACTGGATATTCTTCGAGCAAAAAAGTACATTCTACACTTTCATAAGGTCCTAAAGTAAATGGAATGGAGTAGTTTTTATCTTTATATCGTATAGGTTTGAACGTTTTTTGTTCATTTACTTTATTTTTAATATCAAATTCAACGTCAATAACAGAAATGGGAAACTTTGTGAAATTAATAAATGTTATATCGTTGTAACTTGATTTGTCATCGACCAAGTAATTAAAGCTTCTGGTAGGTATAACATCGATGTTAATAGAATCTTTCATATAGTCTAAATAATATTTAAGTGCAGTCAGTAAGAAACTAAAAATTGCGATACAAATCGCGATTATGTCCATACTTATCACCTCCTTAGGTTGATAACAACATTATACACGAAAGGAGCATAAACAATATGCAAGCATTAAAAACAAAATCGAACATCGGCGAAATGTTCAACATACAAGAAAAAGAAAATGGAGAAATCGCAATAAGTGCAAGAGAGTTATATAAAGCTTTGGAAGTTAAAAAGCGTTTTAGCGCTTGGGCAGAAATTAACTTGAAGCATTTCAAAGAAAATAGGGATTTTACAAGTGTACTTACAAGTACGGTTGTTAATAACGGAGCTGTAAGACAACTAGAAGATTATGCTTTAACACTTGATGTAGCTAAACATGTTGCGATGATGTCAGGTACAGAAAAAGGTTTTGATTTTAGAGAGTATTTCATCCAAGTAGAGAAAGCATGGAACAGTCCAGAAATGATTATGAAACGTGCTTTAAAAATTGCTAACAACACAATCAATCAATTAGAAACAAAGATTGAACGTGATAAACCAAAAATTGTATTTGCAGATGCAGTAGCTACTACTAAGACATCAATTTTAGTTGGAGAGTTAGCAAAGATCATTAAACAAAACGGTATAAACATCGGGCAACGCAGATTGTTTGAGTGGTTACGTCAAAACGGATTCCTTATTAAACGCAAGGGTGTGGATTATAACATGCCTACACAGTATTCAATGGAACGTGAGTTATTCGAAATTAAAGAAACATCAATCACACATTCGGACGGTCATACATCAATTAGTAAGACGCCAAAAGTAACAGGCAAAGGACAACAATACTTTGTTAATAAGTTTTTAGGAGAAAAACAAACATCTTAATAGGAGGAACGAACAATGCAAGCTCAAAACAAAAAAGTCATTTATTACTACTATGACGAAGCCGGTAATAGACGACCCGTTAATATTCAATACAACGATGGCTACGACTTAATGATAGACCCGCGTTTTATTGAAATGACGCTTGAAAGACATCCGCATTTAAAAAATAACTTTTATGGATTAATAGATGGAAAAGAATTTAAGTTAGATTAAATTTTTGGAAATGCAAAGGAGGCATAACAAATGTTACAAAAATTTAGAATCGCGAAAGAAAAAAATAAATTAAAACTCAAATTACTAAAGCATGCTAGTTACTGTTTAGAAAGAAGTAACAACCCTGAATTGTTGCGAGCAGTTGCAGAGTTGTTAAAGAAGGTTAACTAAATTAGGCCTTATTATTACTTTTTAGAATGTGAACAATAGGTCGATAAAAAACTTAATAAACAAACTATAGCAACTATCAATGAATTTTGAATATGTAAATCGTTCTCGTTTATATAGTTTGTTACAAAGATTTGAATGTCAGCACCTGCTGCAATGCCATTAGACCATCTTATTAACTTTTTGAAAGGATGTGGAAAATCATTTTCGATACGTTTGACAAATTCATCGTGTCTCTTGTAGGTACTTTGCTCATTTATTGGATAGGTCGAATTGATGGCTTCAGCCAAAGTAGAGATAGCAGTTGGATTGATATAAAAATCTCTAATGGTCTGTTGTGCTTGAAGTACAATCTCATCATCAAACCTATAGAGTTCCTTAAAAGATTTTATCGTTTCTTCAGAAAATAAATTTCTTTGAAATGTTAGAGATGAAAAAGAATTACGCAAATTAAAATTCATTTCAATTAAGTTGTTTAGATGAAAGTCTACTTTGAAGTCAGAAAATAAATTTATGTTGTTTCTATTAATTATATCTAATTGGTACTTAGGTTTTAAAGATTGTTTAATTGCCATACTTTTAGAAATTTCAACATTACTAATTACGTTATTAATAGAAAAACGAACATTTTTTAAAGGATCAATATACACCAATATCACCTCCTTTCACTAGGAGATAACAACATTATACACGAAAGGAAAGATAGAAATGCCACATATTTTAAACGTAACAGTTCCAATACCTGAAACACATGTACTTATCACAAAAGATGAATATGATGAGCTAATTGGTTATTCATTAGACCCTGTATGGAACATGAGTGACTTAAAGAAGAAATTAAAAATTGCATCTGATGAGACTATCAAGGACAGATTACTATTTCATCCTAGATTTGAAAAAGAACTAAGAGCGCAAGGAATTGTGCATTACCCAGATGAGAATTTTAATCGCTGGAGATTTAACGCAAGAAAGATGAATAAATTCGTCGATGAGCATTTCAATGAAATATATAAGGAGAGAATAAAATGAGCAACATTTATAAAAGCTACCTAGTAGCAGTACTGTGCTTTACAGTCTTAGCAATTGTACTTATGCCGTTTCTATACTTCACTACTGCATGGTCGATTGCGGGATTCGCAAGTATCGCAACATTCATATTTTATAAGGAATACTTTTATGAAGAATAAAAAAACTGTTACTCACGGCAATGAGTAACAGTCTAAACAATTAGAAAATTAATGCATATTCAATATAAAACGAAATAAAGGAAGTGTCAACAATGTACTACAAAATTGGCGATGTATGTCAAAAAGTAATTAATGTAGACGGATTCGATTTTAAATTAGCAGTTAAGAAACAAGATTACAGCATTCTAGTGAATGTCTTAGATTTAGAAGATAGATTTATCGACGGTATAAATATAACAGATGAGAATGATCTATACACAGCATTAGACATATTAAATCAATCTATTTATGAATGGATTGAAGAGAACACAGACGAAAGAGACAGGCTAATTAACTTAGTCATGAGATGGTAGGAGGTTGCTATGAAGCAGACTGTAACTTATCTAATCAAGCATAAAGATGAAAATCTATTTATTACAAACCGACCAACTGAAGTGAACGACACAGTGAAGTATTCAACTGATATGCGAGACGCAAGAGAATTCGACGGACTAGACAAAACCGTTATTGATATGTCTAAGCACAAAGCTATTAAGAAAACAGTGACAGAAACAATTGAGTACGAGAAGGTAGAACATGACTGAAAAAACTAATCAAGATGTCGATATCTTAACGCAACTAGGTGTAAAAGACATCAGCAAACAAAATGCAAACAAGTTTTATAAATTTGCGATATACGGCAAGTTCGGGACTGGTAAAACTACGTTTTTAACAAAAGATAACAACGCCTTAGTACTAGATATAAATGAGGACGGAACAACGGTAACAGAAGATGGGGCAGTTGTGCAGATTAAGAATTACAAGCATTTTAGTGCAGTGATTAAGATGTTACCTAAAATTATTGAACAACTCAGAGAAAACGGAAAACAAATTGATGTTGTAGTGATTGAAACAATCCAAAAGCTACGTGATATCACTATGGACGACATCATGGACGGAAAATTAAAGAAACCAACATTTAATGATTGGGGCGAGTGTGCTACACGCATTGTAAGTATTTATCGTTATATTTCTAAATTACAAGAACATTATCAATTCCATCTTGCTATAAGTGGACACGAGGGAATTAACAAAGACAAAGATGATGAGGGTAGCACTATCAATCCAACAATCACGATAGAGGCACAAGATCAAATAAAAAAAGCGGTCATCAGTCAATCTGATGTGTTAGCAAGAATGACAATAGAAGAACATGAGCAAGACGGCGAAAAAGCTTATCAATATGTTCTTAACGCTGAACCATCAAACTTATTCGAGACAAAGATAAGACACTCAAGCAACATTAAAATTAACAACAAACGTTTCATTAATCCAAGTATTAACGACGTAGTACAAGCAATCAGAAATGGAAACTAATAAAAAAACTAAAAAGGACGGTATTTAATTATGAAAATCACAGGACAAGCGCAATTTACTAAAGAAACAAATCAAGAAAAGTTTTATAACGGCTCAGCAGGGTTTCAAGCTGGAGAATTCACAGTGAAAGTTAAAAATATTGAATTCAATGATAGAGAAAATAGATATTTCACAATCGTATTTGAAAATGATGAAGGCAAACAATATAAACATAATCAATTTGTACCGCCGTATAAATATGATTTCCAAGAAAAACAATTGATTGAATTAGTTACTCGATTAGGTATTAAGTTAAATCTTCCTAGCTTAGATTTTGATACCAATGATCTTATTGGTAAGTTTTGTCACTTGGTATTGAAATGGAAATTCAATGAAGATGAAGGTAAGTATTTTACGGATTTTTCATTTATTAAACCTTACAAAAAGGGCGATGATGTTGTTAACAAACCTATTCCGAAGACAGATAAGCAAAAAGCTGAAGAAAATAACGGGGCACAACAACAAACATCAATGTCTCAACAAAGCAATCCATTTGAAAGCAGTGGCCAATTTGGATATGACGACCAAGATTTAGCGTTTTAAGGTGTGGTTTAAATGCAATACATTACAAGATACCAGAAAGACAATGACGGCACTTATTCCGTCGTTGCTACTGGTGTTGAACTTGAACAAAGTCACATTGACTTACTAGAAAACGGATATCCACTAAAAGCAGAAGTAGAGGTTCCGGATAATAAAAAACTATCTATAGAACAACGCAAAAAAATATTCGCAATGTGTAGAGATATAGAACTTCACTGGGGAGAACCGGTGGAATCAATTAGAAAATTATTACAAACAGAATTGGAAATTATGAAAGGTTATGAAGAAATCAGTCTGCGCGACTGTTCTATGAAAGTTGCAAGGGAGTTAATAGAACTGATTATAGCGTTTATGTTTCATCATCAAATACCTATGAGCATAGAAACAAGCAAGTTGTTAAGTGAAGATAAAGCACTATTGTATTGGGCTACAATCAACCGCAACTGTGTAATTTGTGGAAAGCCTCACGCAGACCTAGCGCATTATGAAGCAGTCGGCAGAGGAATGAACAGAAACAAAATGAATCACTACAACAAACATGTATTAGCGTTATGTCGCGAACACCATAACCAGCAACATGCGATTGGCGTTAAGTCGTTTGATGATAAATATCACTTGCATGACTCGTGGATAAAAGTTGATGAGAGGCTCAATAAAATGCTGAAAGGAGAGAAAAAGGAATGAATAGACTAAGAATAATAAAAATAGCACTCCTAATCGTCATCTTGGCGGAAGAGATTAGAAGCGCTAAAAAAATTAAAAAATTTACCCCTGAGGATTCTAAAGGTTTTCCTGATATAACAAAAGATTCAATAAAAGAACCTAAATAAAAATATTATGGTTGATAAAATCCCATTGTTCTTTTGTTAACCACCCTTGTTTGTTATTGACTATTTCTGTAACAAACAGCTTATCTCCAGAATCGAGATAAGGTTTCAACTTTTCTATCATTTCTGAAGTTGATAAAGAAGAACGGAATAAAAATGAAGATTTCCAATAATTGCAATGACCATTAGAAATTTCCTTTTTTATAACATTTCTCAATTCCTCATATTTTTGTCCGGGTGAGTTTAAATCATATGTTAACATATAAGGTTTTTCCATATTTTATTCACCCCCAATCTAACGCAGTAGCGATAACAAAATTATACCAGAAAGGAGATAACGAAATGGCAACATTTAGAGTTTACAAAGAATCAGGTAACTTTGTCACAGTACACAAAGATTTTATACATGATTCTAATATAAGTTGGAAGGCTAAAGGTATTCTACTTTATTTGTTAAGTCGACCTGATAACTGGCAAATTTACGAAACAGAACTAGAGCAACATTCAACTGATGGACTTAGCGGTTTAAAGAGTGGAATCAAGGAACTGGAAGAAATTGGATACATTCAACGTAGTAGAAAACGTGATAAAAGTGGTAGGTTAAATGGTTATGAGTACTTAGTATATGAGCAACCGCACCACATTCGATTTTCCAACGTTGGAAAAACCGTTAACGGTAAAACCAACAATGGAAAAACCGTTAATGGTAAATCGCATACTACTAATAATAATAGTACTAATAATGATTTAACTAATAATAACAATACTAATAATGAAGGAAGTATATTGTCGGGCAACCCGACGGTGTCTTCCATTCCCTATAAAGAAATTATCGAATACTTAAATAAAAAAGCAGGAAAGCATTTTAAACATAATACAGCTAAAACAAAAGATTTTATTAAAGCAAGATGGAATCAAGATTTTAGGTTGGAGGATTTTAAAAAGGTGATTGATATCAAAACAGCTGAATGGTTAAACACGGATAGCGATAAATACCTTAGACCAGAAACACTTTTTGGCAGTAAATTTGAGGGGTACCTCAATCAAAAAATACAACCAACTGGCACGGATCAATTGGAACGCATGAAGTACGACGAAAGTTATTGGGATTAGGGGGATATTATGAAACCACTATTCAGCGAAAAGATAAACGAAAGCTTGAAAAAATATCAACCTACTCATGTCGAAAAAGGATTGAAATGTGAGAGATGTGGAAGTGAATACGACTTATATAAGTTTGCTCCTACTAAAAAACACCCGAATGGTTACGAGTATAAAGACGGTTGCAAATGTGAAATCTATGAGGAATATAAGCGAAACAAGCAACGGAAGATAAACAACATATTCAATCAATCAAACGTTAATCCGTCTTTAAGAGATGCAACAGTCAAAAACTACAAGCCACAAAATGAAAAACAAGTACACGCTAAACAAACAGCAATAGAGTACGTACAAGGCTTCTCTACAAAAGAACCAAAATCATTAATATTGCAAGGTTCATACGGAACTGGTAAAAGCCACCTAGCATACGCTATCGCAAAAGCAGTCAAAGCTAAAGGGCATACGGTTGCTTTTATGCACATACCAATGTTGATGGATCGTATCAAAGCGACATACAACAAAAATGCAGTAGAGACTACAGACGAGTTAGTCAGATTGTTAAGCGATATTGATTTACTTGTACTAGATGATATGGGTGTAGAGAACACAGAACATACTTTAAACAAACTTTTCAGCATTGTTGATAACAGAGTAGGTAAAAACAACATCTTTACAACTAACTTTAGTGATAAAGAACTAAATCAAAATATGAACTGGCAACGTATCAATTCAAGAATGAAACACAATGCAAGAAAAGTAAGAGTAATCGGAGACGATTTCAGGGAGCGAGACGCATGGTAACCAAAGAATTTTTGAAAATTAAACTTGAGTGTTCAGATATGTACGCTCAGAAACTCATAGACGAGGCACAGGGCGATGAAAATAAGTTATATGACCTATTTATCCAAAAACTTGCAGAACGTCACACACGCCCCGCTGTCGTCGAATATTAAGGAGTGTTAAAAATGCCGAAAGAAAAATATTACTTATACCGAGAAGATGGCACGGAAGATATTAAGGTCATCAAGTATAAAGACAACGTAAATGAAGTTTATTCTCTCACAGGAGCCCATTTCAGCGACGAAAAGAAAATCATGACTGATAGAGACCTAAAACGATTCAAAGGCGCTCACGGGCTTCTATATGAGCAAGAGCTAGGATTACAAGCAACGATATTTGATATTTAGAGGTGGCACAATGAGTAAATACAACGCTAAGAAAGTTGAGTACAAAGGAATTGTATTTGATAGCAAAGTAGAGTGCGAATATTACCAATATTTAGAAAGTAATATGAATGGCACTAACTATGATCGTATCGAACTACAACCTAAATTCGAACTACAACCTAAATTTGGGAAGCAAAGACCGATTACGTATATAGCCGATTTCTCTTTGTGGAAGGAAGGGAAACTGGTTGAAGTTATAGACGTTAAAGGTAAGGCGACTGAAGTTGCCAACATCAAAGCGAAGATATTCAGATATCAGTATAGAGATGTGAATTTAACGTGGATATGTAAAGCGCCTAAATACACAGGTCAAGAATGGATGGTATATGAGGACTTAGTGAAAGTCAGACGTAAAAGAAAAAGAGAAATGAAGTGATTTAATGCAACAACAAGCATATATAAATGCAACGATTGATATAAGAATACCTACAGAAGTTGAATATCATCATTTCGATGATGTGGATGATGAAAAAGATATGCTAGCAAAGCGCTTAGATGACAATCCGGATGAATTACTAAAGTATGACAACATAACAATAAGACATGCATATATAGAGGTGGAATAAATGGCGAAAGCAGCAAGAATTGTAAGGATACACGATAAACCTTATAGGTTCAGTAAATTTGAAATGGAATTAATAGAAAGTCACGGTATAACCGCTGGAATGGTTTCTAAGAGAGTAAAAGACGGTTGGGAACTACATGAAGCAATGGACGCACCAGAAGGTACGCGTTTAAGCGAGTACAGAGAAAAGAAAACAATAGAAAGACTGGAACAAGCTAGACTCGAACGCAAATTGGAAAGAAAGCGAAAGAGAGAGGCTGAGCTAAGAAGAAAGAAGCCACACTTGTTTAATGTACCTCAGAAACATTCACGTGATCCGTACTGGTTTGATAATACTTATAACCAAATGTTCAAGAAGTGGCAGGAAGTATAAATGCCTAAAACCGATAGCGCATGTAAAGAATACTTAAACCAATTTTTCGGCTCTAAGAGATATTTGTATCAGGATAACGAACGAGTGGCACATATCCATGTAGTGAATGGCACTTATTACTTTCACGGGCATATCGTACCAGGCTGGCAAGGCGTGAAAAAGACATTTGATACAACCGAAGAGCTCGAAACATATATAAAGCAACATGGTTTGGAATACGAGGAACAGAAGCAACTAACTTTATTTTAAGGAGATGGAAATAATGAAAATCAAAACTGCAAGCATAGAGGTCGAAAAAGTGGAGGTAGTAGTATGATGCCGAAATTTAGAGCGTGGGATAAAGATAAAAAAGTTATGAGTTTTATTGACGAAATCGATTTTAATAGTGGGTACATTTTGATTTCAACAGGTTATAAAAGTTTCAATGAAGTAAAACTATTACAATACACAGGATTTAAAGATGTGCACGGTGTGGAGATTTATGAGGGGGATATTGTTCAAGATTCTTATTCCGGAGAAGTAAGTTTTATCGAGTTTAAAGAAGGAGCCTTTTATATAACTTTTAGCAATGTAACTGAATTAATAAGTGAAAATGACGATATTATTGAAATTATTGGAAATATTTTTGAAAATGAGGAGCTATTGGAGGTTATGAGATGACGGTCACCTTATCAGATGAACAATATAAAAACCTTTGTACTAAATTAAACAAGTTATTAGGTAAATTTCACAAAGCATTAAAAGAACGTGATGAGTACAAGAAGCAACAAGATGAGCTTATCGTGGATATAGGTAAGTTAAGAGAACGTAACAAAGAGTTGGAGAACATGTGGCGCACTCTTAAAAATGAATTGCTTGGAAGATACGAACATTACTGTTTTAAATTTAGAGAACTACACCCTGAGAGCAAAGCGAACAGGATAGGAGCTCTCTATATAGGAGGTAAAAGCACTGCAGATATTATAATGTCGCGAATGGAAGAACTAGACGGAACAAATGAGTTCTACGAATTTTTAGGGCAAATGGAGGAAGACACAAATGAATAACCGTGAACAAATAGAACAATCCGTTATAAGTGCTAGTGCGTATAACGGCAATGACACAGAGGGATTACTAAAAGAGATTGAGGACGTATATAAGAAAGCGCAAGCGTTTGATGAAATACTTGAGGGAATGACAAATGCTATTCAACATTCAGTTAAAGAAGGTATTGAACTTGATGAAGCAATAGGGATTATGGTAAGTCAAGTTATCTATGAATACAAGGAGGAACTGGAGAATGAAAAAATTTAATGTTCAAATCACATATACAGGCATGATTGAAGAGGCTATCGAGGCTGAAAGTTTAGAAGAAGCAGAATTTGAGGCTCATGATATTGCGAGAATGGAAGTGCCATTTGATTGTGATGAATTTGAAATTAATGTAGAGGTGGAACAGGAAAATGAATAACACATTAACAATTGATCAATTACAAGAGTTATTACAAATACAAAAAGAGTTCGACGATAGAATACCGACGCTGAACTTACGAGATAGCAAGATTGCATATGTAGTTGAATTCTTTGAATGGTTTAATACATTGGAAACGTTTAAGAACTGGAAGAAGAAACCAGGTAAGCCGTTAGACGTACAACTTGATGAATTAGCTGACATGTTGGCGTTTGGGTTGAGTATTGCGAATCAAGTAGGAGTGTCATCAGAAGAGATAAAAGAAGCGATTGAATCAAGTTTTAAAAATACAGAATTTCACAAAATGTTTAATTTTAAAGATAAAGAATTTGCTCAAGACGCAGTTGTTAGTACACCACAGATAATATTCAAAGAATTTTATCCCGACCAATTGGCAATTGTAATAGTGATAGACATAGCTTACAACTTATATTCTATCGACCAACTCATTGACGCATACAAAAAGAAAATGAAAAGGAACCACGAAAGACAAGATGGAACAGCAGACGCAGGAAAAGGATACGTGTAAAGACATCTTAGATCGAGTTAAGGAGGTTTTGGGGAAGTGAGAGAACGCACTAAAATTATATATCGTGGTTGGAACAAGGAGATATTTATTTTACAGGGTAAAAATATGAATGTTATTGGTTTGCGCCAAATATTTGATGAACTCAAAAGATTGTACGAAGGTTATAAAATCGTTGTTATTCCAATAGAAGTTGATTTTGAAATCAAATAAATAGGAGTGATGAGAAGTGACACAATACTTAGTCACAACATTCAAAGATTCATCAGGACTACCACATGAACATTTTACTGCTGCTAGAGATAATCAGACGTTTACAGTTGTTGAGGCGGAGAGTAAAGAAGAAGCGAAAGAGAAGTACGAGGCACAAGTTAAAAGGGATGCAGTTATTAAATTAGGTCAGTTGTTTGAAAATATAAGGGAGTGTGGGAAATGATTAAGCAAATATTAAGATTATTATTCTTACTAGCGATGTATGAGCTAGGTAAGTATGTAACTGAGCAAGTATATATTATGATGACAGCTAATGATGATGTAGAGGCGCCGAGTGATTACGTCTTTCGAGCGGAGGTAAGTGAGTGATGTGGATTACTATGACTATTGTATTTGCTATATTGCTATTAGTTTGTATCAGTATTAATAGTGATCGTGCAAGAGAGATACAAGCACTCAGATATATGAATGATTATCTACTTGATGAAGTAGTTAAAACTAAAGGATACAACGGGTTAGAAGAATACAGGATTGAATTGAAGCGAATAAATAACGATATTAAAAAGTAATTTATATTATCGGAGGTATTGCATGTATAACAGGAAAGAAATACGTGAAATGATAGATAACTACAAGTGGATGAAGAACATAATAGACAGTAAAGTCTACGATAACGAAAGTACATCAATTGCACAATATGGTTATCAATCTGCGATGCCAAAAGCTAAAGGCACGACTAGCAATAAAGTGTTAGTGAAAGTTATAAACAAAAACAAAGCGCTTAGAAAGTACGATTACTTGATTAAGAAGATAGCGTTCATTGATGAATATGAAGAATACATCACGAATGAAAAAGATTATCATATTTTACAAATGTTAAAACAACGAGAAAGCCATAATAGGATCATGAGCATTCTTGATATAGGCAGAGACAATTTTTATTCTAGAGTAAAAGATATAGTAAATATACTTTATAACTTGCAACAAGAAACCGACAGTTCGGACACATCGTACAGTTCGGACACATCGTACAGTTCGGACACATCGTACAGTTCGGACTAATTTTGATGCTACATATTGTTTTTTATTATAATTGCTGTGTAGCAAAACATTTATATTTCTTTTGAACTCTCACATTAAGTGAGGGTTTTTATTTTTATAAACAAGAGGTGGAGAATGGAGATATCAAAGTACCAAGAGATAGCTACACGTACACACAATGATGAATTGAATTTAAATGAATATATTACTTGTTACGGCTTAGGTTTAACTCAATCTACAGGCAATGTTACAGATCTAATTAAACAGCATATGTTTTGTAATGTACCGATAGATAAAGGAATTATGATAAATGAACTTAGCGAAGCATTGTGGAATATAGCTAATCTTACTAACGTGTTAGGTATTAACTTGGATGAGATAGCTGGTCATAGTGTTAACACTATCTTGATGAATAAACCTAATCAGACTATCAATTTAGACAATGGTATAAAACGAGGAGACAAAGTATTGTTTCAAGGTAGTAAGTATCTTGTTGATGGATCGATAGGAAACTTATTGTTAATTAGCAATGATAAAGATGATAGACAAGTAACTGTGCAAGATGTTAAGAAAGTCGACAAGGAGTGATGTGCATTGTCTATTATGAAGCGATGTGGTCATCCAACATGTAATGTATTGATTAATCATAATGAAAGTTATTGTGATAAACACAAGCAATATGCAAATGAAAATTACAATGATTTGAGACGTCGAAACGATCCAGAGTATTTAAGATTTTATAAATCGAAAACGTGGCAAAACATGCGTCGAATTGTATTGTTAGAACATGATTTTATTTGTGTTTCTTGTGGCAATCAAGCGACTATGGTTGACCATATTGTACCAACAAAAATTGATTGGGCAAGAAGATTAGACAAAAGTAATTTACAGCCTTTGTGTGATGCTTGCCATAACCAAAAGACAAAAGAAGATTTGAAGAAATATTAAAAAAGATAAAAATAGGAAGTCACCCCAAAGATGAAACGGGCGTCAATGAAAGGTTCTGGAGAACGGAGCAGAGTTTTCTTCTCAAAAAATTCCCTTTATTTAAGTTTTTTTAGTAGGAGGTGCTAATTTATGGCGGGTAGACCTAAGAAGCTTTTGTCAAATTCGAACAAGAATTATACAAAAGAAGAAATTATTGAAAAAGAGCGTCAAGAAGCTCAATTAAATAAATTTTCTAAAATCGATACTGAACCACCGCACTTTTTAGATGAAATAGCGAAACAAGAATACTTAAGAATATTACCGCACATGCAAGAATTGCCAATTTCCAACTTAGATAAAGCACAATTAGCACAATATTGTAGTTTTTATAGTGACTTTGTTAAAGCAAGTTTGATTTTAGAGCGCGAAGACTTGATTTTAGAAGACGACAAAGGAAATCAAAAGGTTAATCCGGCGTTCAACATAAAGGAAAAAGCGGGTATTCGATTGCAACAAACAGCTAATACTTTAGGATTAACTATTGATAGCCGATTGCGTATTATGGTTCCTGATGAAAAAGAAGATGATGATCCATATATGGAATTTGTGAGTGATTAGTAATGACTGATTATGTTACTAAATACGCAAAAAAGGTAGTTTCAGGAGAAATTTTGGCAAGTTTGAAGAATATTCAAGTATGTAAACGTCACCTATCTTTTATGGAGAACCCGCCGAATGGTTGCCATTGGGATAATCATTTGTCTAACAAAGCAATTAAATTTGTGGAAATGCTTCCAGACCCTAAAACAAACCAGCCCATGCCTCTTATGGAGTTTCAGAAATTCATTGTTGGGAGCTTATACGGCTGGCGTAGAGGTCAATACAGAATGTTTACTAAAGCTTATATAAGTATGGCTAGAAAACAAGGTAAGTCTCTAATCGTATCGGGAATGTCCGTTAACGAACTGTTGTTTGGACAATACCCTAAATTTAATAGACAAATTTATGTAGCTTCATCTACTTATAAGCAAGCGCAAACAATATTCAAGATGGCAAGCCAACAAGTAAACCTAATGCGAAGTAAAAGCAAGTTTATCCGTGAAAAAACAGACGTAAGAAAGACAGACATTGAAGATGTATTAAGTAGTTCAGTGTTTGCACCTCTTTCCAATAACCCAGATGCGGTTGATGGTAAAGATCCTACAGTTGCTATTTTGGACGAATTGGCAAGTATGCCTGATGATGAGATGTACTCAAGGTTTAAAACAGGTATGACATTACAAAAAAATCCTTTAACCCTACTTGTTTCAACGGCCGGAGACAATTTAAATAGTCAAATGTACCAAGAGTATAAGTATATTAAACGTATTTTAAATGAAGAAGTAAGAGCTGATAATTACTTTGTATATTGTGCTGAAATGGATTCACAAGAAGAAGTTCAAGATGAAACAAAGTGGATTAAAGCAATGCCGCTTTTAGAATCAAAAGAACATAGAAAAACTATACTTCAAAATGTAAAAGCTGATATACAAGACGAATTAGAAAAAGGGACATCATATCATAAGATTTTGATTAAAAACTTCAATTTATGGCAAGCGCAAAGAGAAGATAGCTTGCTAGATATTTCAGATTGGGAACAAGTAATAACGCCTATGCCTAATATCAATGGTAAAGATGTGTATATAGGTGTCGACTTATCGAGATTGGATGACTTAACATCTGTAGGGTTTATTTTCCCTAACGACGATAAAAAAGTGTTTTTACATAGTCATTCTTTCATTGGATTAAGAACAAACTTAGAACAAAAATCTAAGAGAGACAAAATAAATTATGAATTAGCGATTGAACGTGGCGAAGCTGAGACTACACAATCAGATAGCGGCATGATTGATTATAAACAAGTTATCGATTTTATAGTGAAATTTATAACGACGCATGACCTGAATGTACAGGCTGTTTGCTATGACCCTTGGAATGCGCAAAGTTTTATAACAACAATCGAATCAATGGCTTTAGATTGGCCACTCATTGAAGTGGGACAAAGTTTTAAGGCGTTATCACAATCTATTAAAGAATTTAGAATGTGGGTTGCAGATGAAAGAATACAGCATAACGATAATATGTTACTTACAACATCAGTTAATAATGCCGTTTTGATTCGTGACGGAGAAGACAATGTGAAAATAAATAAAAAAATGAATCGTCAAAAAATAGATCCGATTATTTCGATTATCACAGCTTTCACTGAAGCTAGAATGCACGAATTCCAAGAAAATTGGACGGAGAAATATGAAAGCGAAGAATTCGGATTTTAAAGGTGGTGACAAAATGGACTTGAATAAAATAAATGTCTTTTTTAATTTCTTGGTTGCTAATTTGGTTAGCATCCTTTTTTTATTAGGTTTGTTTGTGGTTAATGTTTCTGTGTATAAAGCATTCGGTCAAAATATAGGACTTTTATGCATTGGTATAACACTGATTGTTATTTCGTTGATTTTAAATCACGAAAGCAATCAAGAAAGGAGTTAGTAGTTGTGGGGATTTTTTATAAAAATGAAAAACGAGACTTGCAATACAACGAAGATGATTTGCAAATGATGGTTCAAACTTTGCCAGGTTTTCAAGGAACAAAATTACGACAATATAAAGATATAGAAGCAATTAGGCATAGCGACATCTTTACGGCAGTTATGATGATTGCTTCTGATTTGGCGCGCATGCCAATTAGGGTGACAGTGAACGGCCAAATTAATTATAGTGACAGGATTGTTAATTTGTTAAATACACGTCCTAACCCAATGTATAACGGCTATATATTCAAATTAGTAGTGTTTGTTAGTGCCTTACTAACATCGCACGGCTATATTGAAATTACACGTGATAAAACAGGAGAACCTATGAATTTAACGTTCAGAAAGACATCCGAAATAGAATTGAAATCAGACGCAAGAGGTCGACTGTATTATTTTCATCAAAGGATAGACAGTAACGGAAATAATATAGAACGTAATGTTAAGTTTGAGGATATGCTAGACATCAAATTTTATTCGTTGGATGGTATAAATGGTTTGTCACTGTTAGACACATTAAGTCGCACGATAGAATCAGATAACAATGGAAAAGATTTCCTTAATAATTTCTTGCGAAATGGCACACATGCTGGTGGTATTTTGAAAATGAAAGGTGTATTAGATAATAAAAAAGCAAGAGACCGTGCCAGAGAAGAATTTCACAAAAGTTTTAGTGGAACTAAACAAGCTGGGAAAGTTGTCGTACTCGATGAATCAATGACGTTTGATCAATTAGAAGTTGATACAGAAGTTTTAAAGCTTATCAGAGAAAACAAATCATCAACAAGAGAAATAGCAGGTGTATTTGGTATTCCATTGCATAAGTTCGGCATAGAAACAGCGAACATGAGTATCACGGATGCTAATTTAGATTACTTATCAACTTTAAAACCTTATATTACATGCGTTTGTGCAGAATTGAATTTTAAGTTTAATGATGAATATGTGAATCGTGAATTTAAATTTGATACCACTGAAATACGAGTTGTTGATGAAAAAACACAAGCTGAAATTGACAAAATTAACATTGATTCTGGAAAGATGAATATCGATGAAATTAGACAACGTGATGGATTAGCGCCAATACCAGGCGGTAATGGTAGCATTCACAGAGTCGATTTAAACCATGTAAATATTGAACTTGTAGATGAGTATCAGATGAATAAATCGAGAGCTACTGATAAAAAATTGAAAGGTGGTGAGGAAAATGAGTAAGGAAACGAGAGTTGGCAACATTATTGAGGTACGCTCAAATGATAACAACGAAATGGTCATAGAGGGGTATGCGTTAAAGTTTGACACTTGGTCTGAAAATCTTGGTGGATTCAAAGAAACGATTTCACGTCGCGCTTTAGAAAACACTGATTTATCTGATGTGCGTTGTTTAGTAGATCATATCCCATCGCAAATAATTGGTAGGACAAAATCGGGTACTTTGGAGCTCGAAACTGATGATGTTGGACTTAAATATCGTTGTAAGTTACCAAACACAACATTTGCACGTGATTTATATGAGAACATGCGTGTAGGCAACATCAATCAATGTTCGTTTGGTTTTATGCTTGACGATAAAGGCGATGAAGTGCGTTTTGATGAACAAGAAAACATTTACAAACGTACTTTAACAGCAATTCGTGAACTTACAGATGTTTCTGTAGTGACTTATCCGGCTTACAAAGACACTGATGTTAAACCAGCATTACGTAGTATTGAAACCGTTAAAAAAGAACAACGTAAAAAAGAATTAGAAATAAGACTAAAGAAACACTCTATATTAAATAATATTTGGTGAAGTTGAACACCATTATCAAATACAGCCATTGGACATGCTGAATATAGCGATGTCTATTTTTTTATGCCAATTTTAGGAGGAAATTAAATGAAAACAAAAGAAGAGTTACAATCTGAGATTTCAGACATTAAAAGACAAATTGATTTAAAGGTGAAGTATGCAACGAGAGCACTTAATAACGATGAGTTAGAAAAAGCAGAAAAATTAGAACAAGAAATTACTGATTTACGTTCTCAAATCCAAGAAAAACAAGAAGAATTAGATAAGCTAAAAGAAAAAGATGGAACTTCAGAAAACAATCAACAATCAGTGGAAGTAAACGAAGCAAGTACTTATCGAAATCAAGCAAACATTAATGATTTAGGTATTTCGATTCAAAACACAAAGGTAACATCACAAGAAGTTAGAGATTTTACTGAATATCTTGAAACACGCAATGATATTCAAGGTGGTTCGTTAAAAACAGACTCAGGATTTGTAGTTATTCCAGAGGAAATTGTTACAGATATTTTAAAATTAAAAGAGGTTGAGTTTAATCTTGATAAGTATGTGACGGTCAAACGTGTTACAAATGGTTCTGGTAAATATCCGGTAGTACGACAATCAGAAGTTGCAGCCCTTGAAAAAGTTGAAGAATTAGAAGAAAACCCTGAATTAGCAGTTAAACCATTCTTCCAATTAGCATATGACATTAATACACACCGTGGTTACTTCCGAATTTCACGTGAAGCAATCGAAGATGCAAAAGTGAATGTTTTGCAAGAATTGAAACTATGGATGGCGCGAACTATTGCAGCAACACGAAACAAAGCAATTATTGATGTTATCACTAAAGGATCAACGGGTTCTACAAGTTCAGGTTTTGAAAAAGAAGGCAAGAAATTAGAAGTTAAAAAAGCAAAATCTTTAGATGATATTAAAGATGCTATTAACCTGAATGTTAAGCCAAATTACGAACATAATGTTGCGATTGTTTCGCAAACTATGTTTGCAAAATTAGACAAAATGAAAGATAAGCTAGGAAACTATTTAATCCAGCCAGATGTTAAAGAAAAAACGCAACAGCGTTTATTAGGAGCTAAAATCGAAATTTTACCTGATGAAGTACTAGGGCAAAAAGGTAATAACACTTTGATTATCGGTAACTTAAAAGATGCGATTGTTTTATTTGACCGCTCTCAATACCAAGCATCATGGACTGACTACATGCATTTCGGAGAATGTTTAATGATTGCTGTACGTCAAGACTGTAGAATTCTAGATTATAAATCAGCAATTGTGATTGAATATGATGATAGTGAACGCGGTGAAGGCGATCTTGGCTTAGAAGCATAATAAGCGCTCGATACTTTATAAAGAGGTGATAAACTATGGCAATGTATGAAGTGAAGAAATCTTATACTGACTTGGAAAAAGGCCAGTATTTAAAGTCAGGTAAACGTGTTGAAATGACAGTAAAACGTGCTGAATATGTTAACAAAAAGCTGAAAGAGCATGGAGTAATACTTGAAAGAGTAAAAGAAGAATAGGTGATTGAATGCAATTAACAGCTGAGGAACTTAAGTTATTAAAAAAGCATTGCAAAATAGATCACAATTCAGAGGACGACTTATTAGAAATATATTACTCTTGGGCATTCCGTGAAATAGCTAGCGCTGTTACGGATAAACCAAGTAAATATATTGATTGGTTTAAAAGTCATCCTCTATTTGCTCGTGCTATATACCCTTTAGCAAGTTACTATTTTGAAAACCGTATTGCTTATTTGGATAGGGATTTATCGCTTGCGCCACATATGGTTTTAAGTACGGTGCATAAATTGAGAGGTTCATTTGAGCAATTTTTGGAGAGTGAAAATGATGAAATTTAATTCCAATAAATTAAATGAACGTATAGATTTTTGTGAAGATGTAAGCGAGAGAGTGAACGGAAATCCGATGAAACCGAAGACGAAAATATTATACTCTTGTTTCGCTTGCATTCAAGAATCTAAAGAATCCGACACTCAAACGAATCTCAATACAGGTAGCAAATTCATTAAAACTATTATTATCAGAGATACACGAGGTGATTATAAACCAACAAATAAGCATTACGTCTTGCATGAAGGGCAAAGATTTAACATCAAATATGTAAAGCCAGATTATCAAGATAAATCTTATTTGCGTATCTATGGCGAGGTGGTCATTTAATGGGGGCAAGAATTGAAAGTAATAACATCGAACAAGGTTTGAAAAATGCAGTTTTAAAAATGAATTTAAATAGTAATGTAATTGTCAAAGCTGGGGCTATGTCATTAGTCCCGCTTTTAAAAAGTAATACACCTTTTGCGAATACTAAAAAGCATGCTCGCGATCACATAGCTGTTTCTAATGTGAAAACAGACAGACACACAAGTGAGAAAATTGTTACAATTGGTTACGCTAAAGGCGTCTCACATCGTATTCATGCAACAGAATTTGGAACAATGTACCAAAAACCACAATTGTTTATAACAAAAACAGAAAAGCAAGGGAAAAACAAAGTTTTAAAAACAATGCTTGATACTGCTAAGAGGTTGCAAAAATGATTAATGTTACCAAATTAATTAGAAACGCTATTATTGCAAATAACATTACAGATGAAGTGAATGTGTTTAACTACACTATAGATGACCATTTTCACGAAAAAACTGACAAGCCTATTATTCGTATATATCCCTTACCGTTCAATCCTGACACATACGCTGATGATAACGAGATTTCAAGAGAATACCATTACCAAATTGATGTTTGGTGGTCTCAAGATGAACCGAACGAGCAAGCAGAAAAAATTGTTGAGTTACTCAAAGTGATAAATTTTCAATGTTATTACAGAGAACCGTTATACGAGAGTGACGTCATGTCATTCAGACATATTATAAGAGCAAAAGGCTCGATTTTATCAATGAAATTGGAGGAAAATTAAATGATTGAAAAATTGAAACAAGCACCAAGATTTTTAAAATTAAACTTACAACATTTTGCAGATACAGGAGTTTCGGGTATCGCAATTGGGGTATCAAACTTTTATTATGCACCTATTTTAAAAGATACAGAAAATGAATGGGAAACTGGAGCTGGCACACGTATTCGTTTCTTAAAAGAAATTGAAGTAGACCGTCCACAAGATACCGAGGAAGATTATGGGGATGATATGGTCGCAGCAACTGCTGTATCTAATGGCAAACTAAGTGTTAAGACAACATTTGTTACTGTTCCTGCTGACGATAAGGCGTTCTTGAATGGCGCTAAAAAAGGTGTAGGTGGTTATAAATATGGAGCTAAGGATATCCCGCCAGATGTAGCGATTGTATTTGAAAGACGTAATCATGATGAGTCTTCAGAATGGGTTGGCTTGTTCAAAGGTAAATTCACTCGTTCAAGCATCAAAGGGCAAACAAAACAAGATAAAGTTGAATTCCAGAATGACGACGTAGAAGGCAATTTTATTGATCGTTTGTTTGATGAGAGCTCGCATGTTACTGGCTATGATAAAAAAGGAAGCACTACAGGGCGCGATTATGTATTCATGGAAACATTTGGTAAAACTTATGATGAATTCATGTCTAGTCGAGGAGAACAAAATATGGAACCTGTAGAAAAAGAAATGAAAAAAACAGAAAAAGTTGAAGTCACTTCTGTAAACGTCACTGATGAACAAGTTACAGTTAAAGTTGATGCTACTAAACAACTATCAGCCACAACCGAACCATCTGGACAGAAAGTAACTTATGCAGTGACTGAGGGGCAAACGTATGCTAGCGTAACATCAACTGGCCTCGTTAAAGGTTTGGCGGAAGGTAATGCGACCGTTACAGCGACTGCAGGAAAGCAAACTGATACTGTGCAAATTACAGTACAATCTAATTTAGAAATGTAAGTTTTGAGGGCTTAACGCCCTCTTTTTATTTTGGCCAAATTAAAAAGAAAGTAGGAATTTAATAATGGAACGTACATCAATTGAATTAATTACAGGATTTACAAAAACAGGAAAGCCGCAATATCAAAAGTATTTAGCGAAGCCGATTATTACTTTGTTTGAAACAATTCAAGGTTCAAAATTAGGTTTGAAACTTAACAAAGCCTTTAAGGGGGCTGATTTTAAAGATCTAACAGAAGAAGAATTTAATAACTTAAGTGTGACAGAACAGGAAGAATACAAAAACAAGCAAGAAGAATACGAAAACAACATGGCTGTACAAATGGAAGTATTAGAAGAAGTTTTGGATTTCATCGTTGAAGCTTTTGATAATCAATTTACCAGTATAGAACTTCAAAAAGGATTACCAAATGGTCAAGAAGGTATTGAAAAGATTGGACAGTTAATTGGACGAATTACAGGTGGGGAACCTAGCGATACAAAAAAGTTCGTGACAGAGAATCAGAAATAAGAAAAGAAGATTTAACACCTGAAGCTGTCTACAACAATTACAGGAAAATAGCTAAAGATTTGATAGAAAAAGGCATGGATGCAGAAAAAGTGGCTAACATGCCGATACACTTCTTTTTAGACATTGTCGAATCGAAGATTGAAACAAAGCGAACTGCGAAAAGTTTTAAAGATATTTTTTAATCAGCCTTTAAAGGTTGATTTTTTATTTACATCTTGGAAGAAAGGAGGTTTTTAAATGCCTAATCCTATAGGTAATATGGTCATAAAGGTTGATTTAGATGGTTCTGGATTCAATAGAGGTGTGACAGGTTTAAATAGGCAAATGAAAATGGTTTCGCGTGAGCTTTCGGCTAATTTATCACAATTTTCTAGATATGATAATTCATTAGAAAAGTCGAAGATAAAAGTCGAAGGTTTGAGTAAAAAACAAAAAGTTCAAGCCCAGATTACTAAAGAGCTGAAAGATAGTTATGACAAACTTAGTAAAGAAACTGGTGAAAACAGTGCAAAGACACAAGCTGCGGCTGCTAAATACAATGAAGCTTACGCTAAATTAAACCAATATGAGCGAGAGTTAAATCAAGCCACACAAGAATTAAAAGACATGCAAAGAGAGCAGAAAGCATTAAATACTGCAATGGGAAAACTTGGTACCAACTTTAATAATTTTGGTCCTAAACTTCAAGAAATTGGTAACAGTATGAAAAATGTAGGCCGTAACATGACTATGTATGTAACTGCGCCGGTGGTTGCTGGGTTTGCTGTAGCAGCTAAAAAAGGTATTGAATTCGATGACAGTATGAGAAAAGTTAAAGCAACTTCAGGTGCTACTGGGGAAGAGTTTGAAGCTTTGAAGAAAAAGGCTCGCGAAATGGGTGCAACAACAAAATTTAGTGCATCAGATTCGGCTGAAGCATTAAATTACATGGCACTTGCTGGTTGGGATTCTAAGCAAATGATGGAAGGTTTAAGCGGAGTTATGGATTTAGCGGCAGCATCTGGCGAAGAACTGGAAGCAGTAAGTGACATTGTTACAGATGGACTAACGGCATTCGGTTTAAAAGCAAAGGATAGTGGTCATTTTGCGGACATTTTAGCACAAACTAGCTCGAAGGCAAATACGGATGTTAGAGGGCTCGGAGAAGCTTTTAAATATGTCGCTCCTGTAGCAGGTGCGTTAGGTTACACGATTGAAGATACATCTATTGCGATAGGTTTAATGAGTAATGCTGGTATCAAAGGTGAAAAAGCAGGTACAGCGTTACGAACAATGTTCACCAATCTTTCAAGTCCAACTAGAGCTATGGGGAATGAAATGGAACGCTTAGGAATATCTATTACAGATAGTAATGGGAAAATGATTCCTATGCGAAAGCTTTTAGACCAACTGAGGGAAAAATTTAAACATCTTTCAAAAGACCAACAAGCTAGTTCTGCAGCTACAATATTTGGTAAAGAAGCGATGTCAGGAGCATTAGCGATTATAAATGCTTCTGATGAAGACTATCAAAAGTTAACCAAATCTATAGATTCATCTACCGGCGCATCTAAAAGAATGGCCGATACAATGGAATCTGGTTTAGGTGGGAAATTAAGAACTTTAAGGTCGCAATTAGAAGAACTAGCCTTAACGATTTATGACAGAATAGAACCAGCACTAAAGATTATAGTAAGTGCTTTTAGCAAAGTAGTGACATGGGTTACTAAATTACCAACGTCAATTCAATTAGCGGTTGTTGGGTTTGGATTATTTGCAGCAGTTTTAGGTCCTTTAGTTTTTATGTTCGGTTTATTTATCAGCGTGATGGGGAATGCAATGACAGTTTTAGGACCCTTGTTAATAAACGTTAATAAAGCTGGTGGTTTATTCGCGTTTTTAAGAACTAAAATCGCATCACTTGTTAAACTATTTCCGATTTTAGGTGTGTCGATATCAAGTTTAACGTTACCTATAACATTAATTGTAGGTGCATTAGTTGGTATTGGCATAGCTTTCTATCAAGCTTATAAACGTTCAGAAACTTTTAGAAATATTGTAAATCAGGCAATCTCTGGTGTAGCAAACGCATTTAAAGCAGCTAAACTAGCGTTACAAGGTTTCTTTGATTTATTCAAAGGTGATAGTAAAGGCGCGGTTACCCTAGAGAAGATATTTCCACCCGAAACTGTAGCAGGAATACAAAATGTAGTTAATACGATTAGAACAACTTTCTTTAAAGTAGTTGATGCAATCGTTGGTTTCGCCAAAGAGATAGGCGCTCAATTAGCCTCTTTCTGGAAAGAGAACGGCTCAGAAATAACACAAGCTTTGCAAAATATAGCTGGTTTCATTAAAGCAACCTTTGAATTTATTTTTAACTTTATTATTAAACCAATCATGTTTGCGATTTGGCAAGTGATGCAATTTATTTGGCCGGCGGTTAAAGCTTTGATTGTCAGCACTTGGGAAAATATCAAAGGTGTAATACAAGGGGCTATTAATATTATTTTGGGTATTATCAAAGTGTTCTCTAGTCTTTTCACAGGAAACTGGCGAGGCGTTTGGGACGGCATTGTAATGATACTGAAAGGTACTGTGCAGTTAATTTGGAATTTAATACAACTGTGGTTTGTAGGTAAGATTCTAGGTGTTGTTAGATACTTTGGTGGATTGCTTAAAGGTTTAATATCCGGTATCTGGGGTGTTATCAAAGGTATTTTCACAAAATCATTATCTGCAATTTGGAATGCAACGAAAAGTATTTTTGGTTTCTTATACAATAGTGTTAAATCTATTTTCACTAATATGAAAAACTGGTTATCTAGTACGTGGAATAATATCAAAAGCAATACCGTCGGCAAGGCTCATTCGTTATTTACGGGTGTAAGGTCTAAATTCACAAGTTTATGGAATGCGACGAAAGATATATTTACTAAATTAAGAAATTGGATGTCAAACATCTGGAACTCTATTAAAGATAACACGGTAGGTATAGCTGGTCGTTTGTGGGATAAAGTACGTAATATCTTCGGAAACATGCGTGACGGTTTAAAATCTATCATTGGTAAAATTAAAGATCATATCGGCGGTATGGTAGATGCTATTAAAAAAGGACTTAATAAATTAATTGAAGGCTTAAACTGGGTCGGTGGTAAGTTAGGTATGGATGAAATACCTAGGTTACACACTGGTACAGAGCACACACATACTACTACAAGATTAGTTAAGAACGGTAAGATTGCACGTGATACATTCGCTACAGTTGGGGATAAAGGACGTGGAAATGGTCCAAATGGTTTTAGAAATGAAATGATTGAATTCCCTAATGGTAAACGTGTAATCACACCTAGTACAGACACTACTGCTTATTTACCTAAAGGCTCAAAAGTATACAACGGTGCACAAACTTATTCAATGTTAAACGGAACGCTTCCGAGATTTCATTTCGGTACTACTATGTGGAAAGATATTAAATCTAGTGCATCATCGGCATTTAACTGGACAAAAGATCAAATAGGTAAAGGCACAAAGTGGCTTGGCGATAAAGTTGGTGATGTCATGGACTTTATCGATAATCCAGGCAAACTTTTAAATTATGTACTTCAAGCGTTTGGAGTTGATTTCAGTTCTCTAACTAAAGGTATGGGTATTGCTGGCGATATAACAAAAGCTGCATGGTCTAAGATTAAGAAAAGTGCAATCAAGTGGCTTGAGGATGCTTTCGCAGAGTCGGGTGATGGCGGTGTATTAGATATGAGTAAATTACGTTACTTATACGGTCACACTGCTGCTTATACACGAGAAACCGGACGCCCATTCCATGAAGGTCTGGATTTTGATTACATTTACGAACCTGTTCCATCAACCATTAATGGTAGAGCACAAGTTATGCCTGTTCATAATGGTGGTTATGGAAAATGGGTGAAAATTGTAAAGGGCGCCTTAGAAGTTATTTATGCACATTTATCTAAATATAAAGTTAAAACTGGTCAACAAGTTAGGGTCGGACAGACTGTTGGTATATCGGGGAATACGGGGTTTAGTACAGGACCTCACTTACATTATGAGATGCGTTGGAATGGAAGACATAGAGACCCGTTACCGTGGTTAAGAAAGAATAATGGGGGCGGCAAAAGTACACCCGGTGGTAATGGTGCAGCTAATGCTAGACGAGCTATTAAGGCTGCTCAAAATATTTTAGGAGGAAGGTATAAGGCGAGTTGGATTACTAACGAGATGATGCGTGTTGCGAGTCGTGAATCCAATTATACAGCTAATGCAGTCAATAATTGGGATAGCAACGCAAGAGCTGGTATACCTTCAAGAGGTATGTTCCAAATGATAGATCCTTCATTTAGAGCGTACGCAAAGTCGGGTTACAATAATCCTCTCAACCCAACTCATCAAGCTATATCGGCTATGAGATATATTGTGGGTAAATGGGTACCAAGAACAGGCTCATGGAGAGCTGCGTTCAAACGCGCTGGTGATTACGCATATGCTACTGGTGGCAAAGTCTATAACGGATTGTATCACTTAGGGGAAGAAGGATATCCAGAGTGGATAATACCTACTGATCCAAGTAGAGCGAACGAAGCACACAAATTATTAGCTTTAGCTGCTAACGATATTGATAACCGCTCTAAAAATAAGCGACCAAACAACTTACCAAATCCAAGTATAAGTAATAGTGATACAAACTATATTCATACATTGGAGAATAAACTGGATGCGGTTATTAATTGTTTGGTTAGTTTGGTTGAGTCTAATCAAGTTATTGCAGATAAGGATTACGAACCAGTTATTAATAAGTATGTGTTTGAAGATGAGGTAAATAATTCTATCGATAAACGAGAGCGTCACGAATCTACAAGAGTTAGATTTAGAAGAGGAGGCACGATAATCTAATGCAAGATACAATTCAAATAGACAATAAAACAATTGGATGGCTGGTTGTGCAAAGAGGGTTCGAGATACCCTCTTTTAATTTTGTTACTGAAAAAGAAAACGTAAAAGGTAGAGCGGGATCTATTGTTAAGAATCGTTATTTAAATGATATCGAATTTGATTTACCATTAATTATTCGAAACGAAAAATTGTCACCAGGTGGAGAAAAAACACACGATGATATATTAGAAGCATTGGTCAAGTTCTTCAATATTAAAGATTTAACACCTAAAAAACTTAAATTCAAATCTCAAAACTGGTATTGGTTTGCATATTTTGATGGTCCATTAAAATTACCGAAAAACCCAAGAGGTTCAGTGAAGTTCACTATAAAAGTAGTGTTAACAGATCCTTATAAATACTCGGTAACTGGAAACAAAAACACCGCGATTTCAGACCAAGTTTCAGTTGTAAATAGTGGGACTGCTGACACTCCTTTAATTGTTGAAGCCCGAGCAATTAAACCATCTAGTTACTTTATGATCACTAAAAATGATGAAGATTATTTTATGGTTGGTGATGATGAGGTAACCAAAGAAGTTAAGGATTACATGCCTCCTGTTTATCATAGTGAGTTTCGTGATTTCAAAGGTTGGACTAAGATGATTACTGAAGATATTCCAAGTAATGATTTAGGTGGTAAGGTCGGCGGTGACTTTGTGATATCCAATCTTGGCGAAGGATATAAAGCAACTAATTTTCCTGATGCAAAAGGTTGGGTTGGTGCTGGCACGAAACGAGGGCTCCCTAAAGCGATGACAGATTTTCAAATTACCTATAAATGTATTGTTGAACAAAAAGGTAAAGGTGCCGGAAGAACAGCACAACATATTTATGATAGTGATGGTAAGTTACTTGCTTCTATTGGTTATGAAAATAAATATCATGATAGAAAAATAGGACATATTGTTGTTACGTTGTATAACCAAAAAGGAGACCCCAAAAAGATATACGACTATCAGAATAAACCGATAATGTATAACTTGGACAGAATCGTTGTTTATATGCGGCTCAGAAGAGTAGGTAATAAATTTTCTATTAAAACTTGGAAATTTGATCACATTAAAGACCCAGATAGACGTAAACCTATTGATATGGATGAGAAAGAGTGGATAGATGGCGGTAAGTTTTATCAGCGTCCAGCTTCTATCATAGCTATCTATAGTGCGAAGTATAACGGTTATAAGTGGATGGAGATGAATGGATTAGGTTCATTCAATACGGAGATTCTACCGAAACCGAAAGGCGCAAGGGATGTCATTATACAAAAAGGTGATTTAGTGAAAATAGATATGCAAGCAAAAAGTGTTGTCATCAATGAGGAACCAATGTTGAGCGAGAAATCGTTTGGAAGTAATTATTTCAATGTTGATTCTGGGTACAGTGAATTAATCATACAACCTGAAAACGTCTTTGATACGACGGTTAAATGGCAAGATAGATATTTATAGAAAGGAGATGAGAGTGTGATACATGTTTTAGATTTTAACGACAAGATTATAGATTTCCTTTCTACTGATGACCCTTCCTTAGTTAGAGCGATTCATAAACGTAATGTTAATGACAATTCAGAAATGCTTGAACTGCTCATATCATCAGAAAGAGCTGAAAAGTTCCGTGAACGACATCGTGTTATTATAAGGGATTCAAACAAACAATGGCGTGAATTTATTATTAACTGGGTTCAAGATACGATGGACGGCTACACAGAGATAGAATGTATAGCGTCTTATCTTGCTGATATAACAACAGCTAAACCGTATGCACCAGGCAAATTTGAGAAAAAGACAACTTCAGAAGCATTGAAAGATGTGTTGAGCGATACAGGTTGGGAAGTTTCTGAACAAACCGAATACGATGGCTTACGTACTACGTCATGGACTTCTTATCAAACTAGATATGAAGTTTTAAAGCAATTATGTACAACCTATAAAATGGCATTGGATTTTTATATAGAGCTTAGTTCTAATACCGTCAAAGGTAGATATGTGGTACTCAAAAAGAAAAACAGCTTATTCAAAGGTAAAGAAATTGAGTATGGTAAAGATTTGGTTGGGTTAACTAGGAAGATTGATATGTCAGAAATCAAAACAGCATTAATTGCTGTGGGACCCGAAAATGACAAAGGAAAGCGTTTAGAGTTAGTTGTGACTGATGACGAAGCACAAAGTCAATTCAACTTACCTACCCGTTATATTTGGGGAATATACGAACCTCAATCAGATGATCAAAATATGAATGAAACACGGTTGCGTTCTTTAGCCAAAACAGAGTTAAATAAACGTAAGTCGGCAGTTATGTCATATGAGATTACTTCTACTGATTTGGAAGTTACGTATCCGCACGAGATTATATCAATTGGTGATACAGTCAGAGTAAAACATAGAGATTTTAACCCGCCATTGTATGTAGAGGCAGAAGTTATTGCCGAAGAATATAACATAATTTCAGAAAATAGCACATATACATTCGGTCAACCTAAAGAGTTCAAAGAATCAGAATTACGAGAAGAGTTTAACAAACGATTGAACATAATACATCAAAAGTTAAACGATAATATTAGCAATATCAACACTATAGTTAAAGATGTTGTAGATGGTGAATTAGAATACTTTGAACGCAAAATACACAAAAATGATACACCGCCAGAAAATCCAGTCAATGATATGCTTTGGTATGATACAAGTAACCCTGATGTTGCTGTCTTGCGTAGATATTGGAATGGTCGATGGATTGAAGCAACACCAAATGATGTTGAAAAATTAGGTGGTATAACAAGAGAGAAAGCGCTATTCAGTGAATTAAACAATATTTTTATTAATTTATCTATACAACACGCTAGTCTTTTGTCAGAAGCTACAGAATTACTGAATAGCGAGTACTTAGTAGATAATGATTTGAAAGCGGACTTACAAGCAAGTTTAGACGCTGTGATTGATGTTTATAATCAAATTAAAAATAATTTAGAATCTATGACACCCGAAACTGCAACGATTGGTCGGTTGGTAGATACACAAGCTTTATTTCTTGAATATAGAAAGAAATTACAAGATGTCTATACAGATGTAGAAGATGTCAAAATCGCTATTTCAGATAGATTTAAATTATTACAGTCACAATACACTGATGAAAAATATAAAGAAGCGTTGGAAATAATAGCAACAAAATTTGGTTTAACGGTGAATGAAGATTTGCAGTTAGTCGGAGAACCTAATGTTGTTAAATCAGCTATTGAAGCAGCTAGAGAATCCACAAAAGAACAATTACGTGACTATGTAAAAACATCGGACTATAAAACAGACAAAGACGGTATTGTTGAACGTTTAGATACTGCTGAAGCTGAGAGAACGACTTTAAAAGGTGAAATCAAAGATAAAGTTACGTTAAACGAATATCGAAACGGATTGGAAGAACAAAAACAATATACTGATGACCAGTTAAGTGATTTGTCCAATAATCCTGAGATTAAAGCAAGTATTGAACAAGCAAATCAAGAAGCGCAAGAAGCTTTAAAATCATACATTGATGCTCAAGATAATCTTAAAGAGAAGGAATCGCAAGCGTATGCTGATGGTAAAATTTCGGAAGAAGAGCAACGCGCTATACAAGATGCTCAAGCTAAACTTGAAGAGGCAAAACAAAACGCAGAACTAAAGGCTAGAAACGCTGAAAAGAAAGCTAATGCTTATACAGACAACAAGGTCAAAGAAAGCACAGATGCACAGAGGAGAACACTGACTCGCTATGGTTCTCAAATTATACAAAATGGTAAGGAAATCAAATTAAGAACTACTAAAGAAGAGTTTAATGCAACCAATCGTACACTTTCAAATATATTAAACGAGATTGTCCAAAACGTTACAGATGGAACAACAATCAGATATGATGATAACGGAGTGGCTCAAGCTTTAAATGTGGGGCCACGTGGTATTAGATTAAATGCTGATAAAATTGATATTAACGGTAATAGAGAAATAAACCTTCTTATCCAAAATATGCGAGATAAAGTAGATAAAACCGATATTGTCAACAGCCTTAATTTATCAAGAGAGGGTCTTGATATCAATGTTAATAGAATTGGAATTAAAGGCGGTAACAATAACAGATATGTTCAAATACAGAATGATTCTATTGAACTAGGTGGTATTGTGCAACGAACTTGGAAAGGCAAACGATCAACCGATGATATATTCACACGTCTTAAAGATGGACATCTAAGGTTTAGAAATAATACCGCAGGCGGTTCACTTTATATGTCACATTTTGGTATTTCAACATATATTGATGGAGAAGGCGAAGACGGAGGTTCATCCGGTACTATTCAATGGTGGGATAAAACTTACAGTGATAGCGGTATGAATGGCATAACAATCAATTCCTATGGTGGTGTCGTTGCACTAACGTCAGATAATAATCGGGTTGTTCTGGAGTCTTACGCTTCATCGAATATCAAAAGCAAACAGGCACCGGTGTATTTATATCCAAACACAGACAAAGTGCCTGGATTAAACCGATTTGCATTCACGCTGTCTAATGCAGATAACGCTTATTCGAGTGATGGTTATATTATGTTTGGTTCTGATGAGAACTATGATTACGGTGCGGGTATCAGGTTTTCTAAAGAAAGAAATAAAGGTCTTGTTCAAATTGTTAATGGACGATATGCAACAGGTGGAGATACAACAATCGAAGCAGGGTATGGCAAATTTAATATGCTGAAACGACGTGATGGTAATAGGTATATTCATATACAGAGTACAGACCTACTGTCTGTAGGTTCAGATGATGCAGGAGATAGGATAGCTTCTAACTCAATTTATAGACGTACTTATTCGGCCGCAGCTAATTTGCATATTACTTCTGCTGGCACAATTGGGCGTTCGACATCAGCGCGTAAATACAAGTTATCTATCGAAAATCAATATAACGATAGAGATGAACAACTGGAACATTCAAAAGCTATTCTTAACTTACCTATTAGAACGTGGTTTGATAAAGCTGAGTCTGAAATTTTAGCTAGAGAGCTGAGAGAAGATAGAAAATTATCGGAAGACACCTATAAACTTGATAGATACGTAGGTTTGATTGCTGAAGAGGTGGAGAATTTAGGATTAAAAGAGTTTGTCACGTATGATGACAAAGGAGAAATTGAAGGTATAGCGTATGATCGTCTATGGATTCATCTTATCCCTGTTATCAAAGAACAACAACTAAGAATCAAGAAATTGGAGGAGTCAAAGAATGCAGGATAACAAACAAGGATTACAAGCTAATCCTGAATATACAATTCATTATTTATCACAGGAAATTATGAGGTTAACACAAGAAAACGCGATGTTAAAAGCGTATATACAAGAAAATAAAGAAAATCAACAATGTGCTGAGGAAGAGTAATCCTTAGCACTATTTTTATACAAAAATTTAAGGAGGTCATTTAATTATGGCAAAAGAAATTATCAACAATACAGAAAGGTTTATTTTAGTACAAATCGACAAAGAAGGTACAGAACGTGTAGTATATCAAGATTTCACAGGAAGTTTTACAACTTCTGAAATGGTTAACCATGCTCAAGATTTTAAATCTGAAGAAAACGCTAAGAAAATTGCGGAGACGTTAAATTTGTTATATCAATTAACTAACAAAAAACAACGTGTGAAAGTAGTTAAAGAAGTAGTTGAAAGATCAGATTTATCTCCAGAGGTAACAGTTAACACTGAAACAGTATGAAAAGCTATGAGTTAGATACTCATAATCTTTATTCTTTTAGAAAGCGGGTGTACTGAATTGGGGTGGTTCAAAAAACACGAACATGAATGGCGCATCAGAAGGTTAGAAGAGAATGATAAAACAATGCTCAGCACACTCAACGAAATTAAATTAGGTCAAAAAACCCAAGAGCAAGTTAACATTAAATTAGATAAAACCTTAGATGCTATTCAAAAAGAAAGAGAAATAGATGAAAAGAATAAGAAAGAAAATGATAAGAACATACGTGATATGAAAATGTGGGTGCTTGGTTTAGTTGGGACAATATTTGGGTCGCTAATTATAGCATTATTGCGTATGCTTATGGGCATATAAGAGAGGTGAATAAAATGTTTAAACTAATCTTTGGTTATAGTTTCTGGACATGTTTTTGGTTCGGTAAATGTAAATAAGTTTTAGTCAGTGCTTCGGTACTGACTTTTTATTTATTGTTGTAATTATGGTAATATGCAGAAGTGAGCAAGTTGGATAGATGGTGGCTATCTGAGTATAAGGAGGTGGTGCCTATGGTGGCATTACTGAAATCTTTAGAAAGGAGACGCCTAATGATTACAATTAGTACCATGTTGCAGTTTGGTTTATTCCTTATTGCATTGATAGGTCTAGTAATCAAGCTTATTGAATTAAGCAATAAAAAATAACCATCGCTAACTTTGGCTGGTTTCGATGGTTAAATGGTTATTAATTTAATCTTTAATCTAAAATAGCCACCGTCTTTTTAACGGGCTCATTAGGGTAACATGTTTGCGCATGTTGCCCTTTTTCTATATATAAATTAACACACCATAATATAAATATCAAATAGACGGCTTATTAGTCGTCTTTTTATTTTGGGTAAAAGGAGATAAGAATATGATTAATTGGAAAATTAGAATGAAACAAAAATCATTTTGGGTAGCGATATTGTCAGCTATCTTTTTATTTGCTCAAAACATCGCAAAAGCTATTGGGTATGATATCCAAGTTTATACAGAGCAATTAACAGACGGTTTAAACGCTATATTAGGATTTTTAGTATTAACTGGTGTGATTCAAGACCCGACTACTAAAGGTATAGGTGATAGCCACCAAGCTTTAGAATATGAAGAACCAAGAAGAAAATACTAGGAGGTAAAATAATGAAAACATACAGTGAAGCAAGAGCAAGGTTACGTTGGTATCAAGGTAGATATATTGATTTTGACGGTTGGTATGGTTACCAATGTGCAGATTTAGCAGTTGATTACATTTATTGGTTGTTAGAAATTAGAATGTGGGGAAATGCAAAAGATGCAATCAATAACGATTTTAAAAACATGGCAACAGTATATGAAAACACACCATCGTTTGTTCCACAAATAGGTGATGTGGCTGTATTTACCAAAGGAATATATAAACAATACGGTCATATTGGTTTAGTGTTTAATGGTGGTAATACAAACCAATTTTTAATTTTGGAACAGAACTATGACGGTAACGCAAATACGCCTGCAAAGTTACGTTGGGATAATTATTACGGCTGTACTCACTTTATTAGACCTAAGTATAAAAGTGAGGGCTTAATGAATAAGATCACAAATAAAGTTAAACCACCTGCTCAAAAAGCAGTCGGTAAATCTGCAAGTAAAATAACAGTTGGAAGTAAAGCGCCTTATAACCTTAAATGGTCAAAAGGTGCTTATTTTAATGCGAAAATCGACGGCTTAGGTGCTACTTCAGCCACTAGATACGGTGATAATCGTACTAACTATAGATTCGATGTTGGACAGGCTGTATACGCGCCTGGAACATTAATATATGTGTTTGAAATTATAGATGGTTGGTGTCGCATTTATTGGAACAATCATAATGAGTGGATATGGCATGAGAGATTGATTGTGAAAGAAGTGTTTTAATTCTTAGGTTAAAATGTTAAATATTTGTTAATTATTTTTTAATGTAAGTTTAGTTTCTTTTAATATTTTATTGATTTTTAATATTTTTTCGATATAAAATGAAGTTGTTGATATTTATCATCTTAAATAAGGGTGTTAGCTATAAAAAGAGATAAATAAAAACAAATATATTATATTTGGAGGAAGCGCCATGCTCAAAAGAAGTTTATTATTTTTAACTGTTTTATTGTTATTATTCTCATTTTCTTCAATTACTAATGAGGTAAGTGCATCAAGTTCATTCGACAAAGGAAAATATAAAAAAGGCGATGACGCGAGTTATTTTGAACCAACAGGCCCGTATTTGATGGTAAATGTGACTGGAGTTGATGGTAAAGGAAATGAATTGCTATCCCCTCATTATGTCGAGTTTCCTATTAAACCTGGGACTACACTTACAAAAGAAAAAATTGAATACTATGTCGAATGGGCATTAGATGCGACAGCATATAAAGAGTTTAGAGTAGTTGAATTAGATCCAAGCGCAAAGATCGAAGTCACTTATTATGATAAGAATAAGAAAAAAGAAGAAACGAAGTCTTTCCCTATAACAGAAAAAGGTTTTGTTGTCCCAGATTTATCAGAGCATATTAAAAACCCTGGATTCAACTTAATTACAAAGGTTATTATAGAAAAGAAATAAAACAAAATAGTTGTTTATTATAGAAAGCAATGTCTTGATTGAATATGTGTAGTGAAAATTATCTTTCATCAAATTCTCATTCATGCACGAATGGTTCTTCCCCACCTAATCAGATATTAGGTGACTTATGGGGAGAAATCAGTTAGGATGAAAAAGTGGATAATCCTTTTTTAGGCAGGTACTTCGGTACTTGCCTATTTTTTTATGTTATAATCTTTCTAGACGTATTCAAGGGACGTCTTTTTAGATTGTATGTTATAGCTAGCTTTCGGGCTAGTTTTTTGTTATGATGTGTTACACATGCATCAACTATTTACATCTATCCTTGTTCACCCAAGCATGTCACTGGGTGTTTTTTCTTATGATAGAGAGCATAGTTTTCATACTACTCCCTCGTAGTATATATGACTTTAGCATTCCCGTATAATAGTTTACGGGGTGCTTTTTATGTTATAATTAACTGTATATAGTAGGAGTGAACTATATAGCCTGTTAAGTGGCCTAGTAACCTAACACTTATCCTGCAATTGATATCCTTTTTGCCCTTCACTCGATACATATATCTCAACAACATAGAAATATTACAGTCGCTACACCGCATCTTAAATGGTGTGGTTATTTTTATTGGAAGTGTGTATCAGGTATCAGTAATGTTAAAACACCAGCTAAAAATGAAAAGAATTCACCAGTGCCAGCAGGTTATACACTCGATAAAAACAATGTACCGTATAAAAAAGAGACTGGTTATTACACAGTTGCCAATGTTAAAGGTAATAACGTGAGGGATGGCTATTCAACTAATTCAAGAATTACAGGTGTATTACCCAATAACGCAACTATCAAATATGACGGCGCATATTGCATTAATGGCTATAGATGGATTACTTATATTGCTAATAGTGGACAACGTCGTTATATAGCGACAGGAGAGGTAGACAAGGCAGGTAATAGAATAAGCAGTTTTGGTAAGTTTAGTGCAGTTTGATAATTAGATATATAAAGGTTTGGCAAGTTATGAAATGTCTGCCAAACCTTTATATAAAAAAGAAATATCTACCTTTTAATCCGAGGTATGAAAACGAGAATTGGACCTTTACAGAATTACTCTATGAAGCGCCATATTTAAAAAGCTACCAAGACGAAGAGGATGAAGAGGATGAGGAGGCAGATTGCCTTGAATATATTGACAATACTGATAAGATAATATATCTTTTATATAGAAGATATCGCCGTATGTAAGGATTTCAGGGGGCAAGGCATAGGCAGCGCGCTTATCAATATATCTATAGAATGGGCAAAGCATAAAAACTTGCATGGACTAATGCTTGAAACCCAGGACAATAACCTTATAGCTTGTAAATTCTATCATAATTGTGGTTTCAAAATCGGCTCCGTCGATACTATGTTATACGCCAACTTTCAAAACAACTTTGAAAAAGCTGTTTTCTGGTATTTAAGGTTTTAGAATGCAAGGAACAGTGAATTGGAGTTCGTCTTGTTATAATTAGCTTCTTGGGGTATCTTTAAATACTGTAGAAAAGAGGAAGGAAATAATAAATGGCTAAAATGAGAATATCACCGGAATTGAAAAAACTGATCGAAAAATACCGCTGCGTAAAAGATACGGAAGGAATGTCTCCTGCTAAGGTATATAAGCTGGTGGGAGAAAATGAAAACCTATATTTAAAAATGACGGACAGCCGGTATAAAGGGACCACCTATGATGTGGAACGGGAAAAGGACATGATGCTATGGCTGGAAGGAAAGCTGCCTGTTCCAAAGGTCCTGCACTTTGAACGGCATGATGGCTGGAGCAATCTGCTCATGAGTGAGGCCGATGGCGTCCTTTGCTCGGAAGAGTATGAAGATGAACAAAGCCCTGAAAAGATTATCGAGCTGTATGCGGAGTGCATCAGGCTCTTTCACTCCATCGACATATCGGATTGTCCCTATACGAATAGCTTAGACAGCCGCTTAGCCGAATTGGATTACTTACTGAATAACGATCTGGCCGATGTGGATTGCGAAAACTGGGAAGAAGACACTCCATTTAAAGATCCGCGCGAGCTGTATGATTTTTTAAAGACGGAAAAGCCCGAAGAGGAACTTGTCTTTTCCCACGGCGACCTGGGAGACAGCAACATCTTTGTGAAAGATGGCAAAGTAAGTGGCTTTATTGATCTTGGGAGAAGCGGCAGGGCGGACAAGTGGTATGACATTGCCTTCTGCGTCCGGTCGATCAGGGAGGATATCGGGGAAGAACAGTATGTCGAGCTATTTTTTGACTTACTGGGGATCAAGCCTGATTGGGAGAAAATAAAATATTATATTTTACTGGATGAATTGTTTTAGTACCTAGATTTAGATGTCTAAAAAGCTTTAACTACAAGCTTTTTAGACATCTAATCTTTTCTGAAGTACATCCGCAACTGTCCATACTCTGATGTTTTATATCTTTTCTAAAAGTTCGCTAGATAGGGGTCCCAAAAGATTTATAACGAAATTGACGAAGCACTAAAAAGTAAATATTAAAAAAACCACCCTTTTACGGGTGGTTTTAATTTTCTAGATAATATAAAAGTGTTCATAAATAAAACAGTATAGGCAAACAATAAAGTATTGAAAAAAGTAAGTTTAATATGAAAATTGTTAAATGAACGACATCTTTTGTTTTTATAAATATCAAGAAAATAATCAAACTCAAAATAAATAACGTAACTGTAGTCATAGGCGTCCATACATAATCAGCATTAGTCATTAAGAATGGTGCAGCCATTATGAAAAAATTTATAATGCAGATGAAATAGACAATTAGACTATAAATTAGGTAAATAACAATACACACCCTTCATAAATAAATAATTTAAATCCTATATATTTTAACAAAAGTAAAACACAGAAGTGTAGAAAATAAAAAATATTGGTAAATAAAATCAATAAGTTTAACCAATATGTTGCTCGCTTCATACCGTATATTGCAACAAAAATTCCGATCAAGAAAAATATAGCCCCTATGATAAAACAGAAATCCGATGCTGAACTATTAAAAAATGAGGTGTTTAGAGTTAGAAAATGAGTTAATGAGTTGACTATAACTAATAAGATATTAATTATATTTGTATGGTTCTTCACATGATACCTCCAAGTAAAAAAATCTAATTAATAAAGTGAATGCTTGATGAACAAGCAGTTATTCCAAACAGAATCAATAAGAAAAGTAGAATCAACATGCTAATGCCCCATAAACAACCCTTTTCACTTTCTCTATTATTAATTTCTTGACTTCTTTTTAAAGATTTATTACTTTTACATTCTTTAGTTGTTTTAAATTTCACGTTTTTATTACTTCCTTTTGTCTAAAAGTTTACAATGAATTTTTGATTATAATAATATATTCAAAATAGTACTATCTAGTTTGATATGTCAAGCAATATTATTATAAAATTGGAATTCTGAGTTGTCTACTCTAATTTATTATATTTACCTATAAAAATACACCTCAAAAAATAGATTTTTCAGTCTAGCTTTTGGGGTGTACATTCCACACAAACATGTGATTATTTTGATGTTTCTATTAAACTTGTAATTTTAAATTTAAAGTCCCTAAAAAGTCCCTAAAATTTTATTTTATATGGGGTATTATTGATAATGATAAAGTTATAAACCTTGATATTATGCTGTTTTACTTTTTGAATGATAAGTAATTTTATGTTAAAAGTCT**

**Supplementary tables**

**Table S1: Strains**

| Strain | Description | Reference/ Origin |
| --- | --- | --- |
| *Escherichia coli* |  |  |
| DC10B |  | (1) |
| *Staphylococcus aureus* |  |  |
| 8325-4 (RN0450) | NCTC8325 cured of Φ11, Φ12 and Φ13 | (2) |
| 8325-4 Φ13K | Single-lysogen, *kan^R^* | (3) |
| 8325-4 Φ13K-*rep* | Single-lysogen, carrying replication deficient phage mutant (3), *kan^R^* | (3) |
| SH1000 | *rsbU* repaired derivative of 8325-4 | (4)  Susanne Engelmann, TU Braunschweig, Germany |
| SH1000 Φ13K | Single-lysogen, *kan^R^* | (3) |
| SH1000 Φ13K-*rep* | Single-lysogen, carrying replication deficient phage mutant (3), *kan^R^* | (5) |
| Newman-c | Phage-cured | (6) |
| Newman-c Φ13K | Single-lysogen, *kan^R^* | (3) |
| Newman-c Φ13K-*rep* | Single-lysogen, carrying replication deficient phage mutant (3), *kan^R^* | This study |
| MW2c | Phage-cured | (7) |
| MW2c Φ13K | Single-lysogen, *kan^R^* | (3) |
| MW2c Φ13K-*rep* | Single-lysogen, carrying replication deficient phage mutant (3), *kan^R^* | (3) |
| SH1000 Φ13K-*TATA* | Single-lysogen, *p23* TATA-Box substitution, *kan^R^* | (5) |
| Newman-c Φ13K-*TATA* | Single-lysogen, *p23* TATA-Box substitution, *kan^R^* | (5) |
| SH1000 Φ13K-*ltr* | Single-lysogen, carrying *ltr* deficient phage mutant, *kan^R^* | This study |
| Newman-c Φ13K-*ltr* | Single-lysogen, carrying *ltr* deficient phage mutant, *kan^R^* | This study |
| RN4220-331 | *ΔmazEFrsbUVWsigB*::*tetM* | (8) |
| 8325-4 Φ13K *sigB* | Single-lysogen, *sigB*::*tetM* | This study |
| SH1000 Φ13K *sigB* | Single-lysogen, *sigB*::*tetM* | This study |
| Newman-c Φ13K *sigB* | Single-lysogen, *sigB*::*tetM* | This study |
| SM2 | Newman *spoVG*::*erm*, *yabJ-spoVG* mutant, *erm^R^* | (9)  Markus Bischoff |
| SH1000 Φ13K *spoVG* | Single-lysogen, *spoVG*::*erm* | This study |
| LS1 |  | (10)  Löffler, Münster, Germany |
| RN4220 | restriction deficient derivate of 8325-4, rK-mK+ | (11) |

**Table S2: Oligonucleotides**

| Oligonucleotide | Sequence | Used for |
| --- | --- | --- |
| pCG896gibfor | gctggcggccgctgcatgGGATCAT  GAGCATTCTTGATATAGGC | Cloning pCG896 |
| pCG896gibrev | cataaataatcatcctcctaagCCCTC  ACTTAATGTGAGAGTTCA | Cloning pCG896 |
| pcIyfpoutsidecontrolfor | GGACAGGTATCCGGTAAGCG | Cloning |
| pcIyfpcontrolrev | TGACAAGTGTTGGCCATGGA | Cloning |
| pCG896insidecontrolfor | GGACACATCGTACAGTTCGG | Cloning |
| pCG910SDMfor | cggccGCTGTGTAGCAAAACATTTA  TATTTC | Cloning pCG910, pCG925 |
| pCG910SDMrev | cggcgAAAAACAATATGTAGCATCA  AAATTAG | Cloning pCG910, pCG925 |
| pCG943SDMfor | TAATTTTGATGCTACATATTGTTTT  TTATTATAATTG | Cloning pCG943 |
| pCG943SDMrev | CGGTTTCTTGTTGCAAG | Cloning pCG943 |
| pIMAYcontrolfor | CCAGCCCCCTCACTACAT | Cloning pCG925, pCG926 |
| pIMAYcontrolrev | ATCACCCGACGCACTTTG | Cloning pCG925, pCG926 |
| pCG925gibfor | aattcctgcagcccggggCTATGACTATT  GTATTTGCTATATTGCT | Cloning pCG925 |
| pCG925gibrev | gccgctctagaactagtgGCACATCACTC  CTTGTCGAC | Cloning pCG925 |
| pCG925outsidecontrolfor | GCGGAGGTAAGTGAGTGA | Cloning pCG925 |
| pCG925outsidecontrolrev | GGATGACCACATCGCTTCA | Cloning pCG925 |
| pCG926insert1gibfor | aattcctgcagcccggggAGACATC  TTAGATCGAGTTAAGGAGG | Cloning pCG926 |
| pCG926insert1gibrev | ttcctgtttTACATGCAATACCT  CCGATA | Cloning pCG926 |
| pCG926insert2gibfor | ttgcatgtaAAACAGGAAAGAAA  TACGTGA | Cloning pCG926 |
| pCG926insert2gibrev | gccgctctagaactagtgAATAGACA  ATGCACATCACTCCT | Cloning pCG926 |
| 926outsidecontrolfor | CGACCAACTCATTGACGC | Cloning pCG926 |
| 926outsidecontrolrev | AACCATAGTCGCTTGATTGCCACA | Cloning pCG926 |
| circlefor | TTTTATTTTATATGGGGTATTATTGA | qPCR (Φ13) |
| circlerev | GTGTATTCTCATTTGTTAGAAGAAAA | qPCR (Φ13) |
| SAOUHSC_02200qPCRfor | GGCACGACTAGCAATAAA | RT-qPCR (*ltr*) |
| SAOUHSC_02200qPCRrev | GTCTCTGCCTATATCAAGAAT | RT-qPCR (*ltr*) |
| cIqPCRfor | AGAACGTCAAGATGAAACGA | RT-qPCR (*cI*) |
| cIqPCRrev | AATTCTTCTCCTATGCCAGC | RT-qPCR (*cI*) |
| SAOUHSC02234DIGfor | TAATACGACTCACTATAGGGAG  ATGCAAAATTGTACTGAGTGC | RT-qPCR (*mor*) |
| SAOUHSC02234DIGrev | ATGTGTTACGACTACTCACG | RT-qPCR (*mor*) |
| SAOUHSC02196for2 | CACGAATCAAAACGGCATTA | RT-qPCR (*terL*) |
| SAOUHSC02196rev2 | ACAACAATCGAATCAATGGC | RT-qPCR (*terL*) |
| SAOUHSC02191for | TTTGCATCTTCGATTGCTTC | RT-qPCR (*mcp*) |
| SAOUHSC02191DIGrev | TACGACAATCAGAAGTTGCA | RT-qPCR (*mcp*) |
| RTqPCRamidasefor | AAATAGGTGATGTGGCTGTA | RT-qPCR (*amidase*) |
| RTqPCRamidaserev | AGTGAGTACAGCCGTAATAATT | RT-qPCR (*amidase*) |
| holin255for | ATGATTAATTGGAAAATTAGAA | RT-qPCR (*holin*) |
| holin255rev | CTAGTATTTTCTTCTTGGTTCT | RT-qPCR (*holin*) |
| sakLClo | CATCAAGTTCATTCGACAAAGGAAA | RT-qPCR (*sak*) |
| sak-A | TGTAGTCCCAGGTTTAATAGG | RT-qPCR (*sak*) |
| asp493f | AAAATTGCTGGTATCGCTGC | RT-qPCR (*asp*) |
| asp848r | TGTAAACCTTGTCTTTCTTGGT | RT-qPCR (*asp*) |
| T7-SAOUHSC02196DIGfor | TAATACGACTCACTATAGGGAG  ATTAGGGTCTGGAAGCATTTC | DIG-probe Northern Blot (*terL*) |
| SAOUHSC02196DIGrev | ATGGTTGCCATTGGGATAAT | DIG-probe Northern Blot (*terL*) |
| T7-SAOUHSC02191DIGfor | TAATACGACTCACTATAGGGA  GATTTGCATCTTCGATTGCTTC | DIG-probe Northern Blot (*mcp*) |
| SAOUHSC02191DIGrev | TACGACAATCAGAAGTTGCA | DIG-probe Northern Blot (*mcp*) |

**Table S3: Plasmids**

| Plasmid | Description | Resistance casette | Reference/ Origin |
| --- | --- | --- | --- |
| pIMAY-Z | Mutagenesis vector | *cm* | (12) |
| pCG725 | P*_cap_*-*yfp* | *cm* | (8) |
| pCG896 | Promoter construct P_23_-*yfp* | *cm* | This study |
| pCG910 | Promoter construct P_23_-*TATA*-*yfp* | *cm* | This study |
| pCG925 | Mutagenesis vector for p23 TATA Box mutation (pIMAY-Z) | *cm* | (5) |
| pCG926 | Mutagenesis vector for *ltr* mutation (pIMAY-Z) | *cm* | This study |
| pCG943 | Promoter construct P_23_-Δ*repeat*-*yfp* | *cm* | This study |

**References**

1. Monk IR, Shah IM, Xu M, Tan MW, Foster TJ. Transforming the untransformable: application of direct transformation to manipulate genetically Staphylococcus aureus and Staphylococcus epidermidis. mBio. 2012;3(2).

2. Novick R. Properties of a cryptic high-frequency transducing phage in Staphylococcus aureus. Virology. 1967;33(1):155-66.

3. Rohmer C, Dobritz R, Tuncbilek-Dere D, Lehmann E, Gerlach D, George SE, et al. Influence of Staphylococcus aureus Strain Background on Sa3int Phage Life Cycle Switches. Viruses. 2022;14(11).

4. Horsburgh MJ, Aish JL, White IJ, Shaw L, Lithgow JK, Foster SJ. sigmaB modulates virulence determinant expression and stress resistance: characterization of a functional rsbU strain derived from Staphylococcus aureus 8325-4. J Bacteriol. 2002;184(19):5457-67.

5. Dobritz R, Rohmer C, Niepoth E, Egle V, Korn N, Bisanzio V, et al. Multiple effects of the bacterial DNA-binding protein SarA on the life cycle of Staphylococcus aureus phages. J Bacteriol. 2025:e0027925.

6. Bae T, Baba T, Hiramatsu K, Schneewind O. Prophages of Staphylococcus aureus Newman and their contribution to virulence. Mol Microbiol. 2006;62(4):1035-47.

7. Tang Y, Nielsen LN, Hvitved A, Haaber JK, Wirtz C, Andersen PS, et al. Commercial Biocides Induce Transfer of Prophage Φ13 from Human Strains of Staphylococcus aureus to Livestock CC398. Front Microbiol. 2017;8:2418.

8. Keinhörster D, Salzer A, Duque-Jaramillo A, George SE, Marincola G, Lee JC, et al. Revisiting the regulation of the capsular polysaccharide biosynthesis gene cluster in Staphylococcus aureus. Mol Microbiol. 2019;112(4):1083-99.

9. Meier S, Goerke C, Wolz C, Seidl K, Homerova D, Schulthess B, et al. sigmaB and the sigmaB-dependent arlRS and yabJ-spoVG loci affect capsule formation in Staphylococcus aureus. Infect Immun. 2007;75(9):4562-71.

10. Bremell T, Abdelnour A, Tarkowski A. Histopathological and serological progression of experimental Staphylococcus aureus arthritis. Infect Immun. 1992;60(7):2976-85.

11. Kreiswirth BN, Lofdahl S, Betley MJ, O'Reilly M, Schlievert PM, Bergdoll MS, et al. The toxic shock syndrome exotoxin structural gene is not detectably transmitted by a prophage. Nature. 1983;305(5936):709-12.

12. Monk IR, Tree JJ, Howden BP, Stinear TP, Foster TJ. Complete Bypass of Restriction Systems for Major Staphylococcus aureus Lineages. mBio. 2015;6(3):e00308-15.
